# Genome resequencing reveals independent domestication and breeding improvement of naked oat

**DOI:** 10.1093/gigascience/giad061

**Published:** 2023-08-01

**Authors:** Jinsheng Nan, Yu Ling, Jianghong An, Ting Wang, Mingna Chai, Jun Fu, Gaochao Wang, Cai Yang, Yan Yang, Bing Han

**Affiliations:** Key Laboratory of Germplasm Innovation and Utilization of Triticeae Crops at Universities of Inner Mongolia Autonomous Region, Inner Mongolia Agricultural University, Hohhot 010010, China; Key Laboratory of Germplasm Innovation and Utilization of Triticeae Crops at Universities of Inner Mongolia Autonomous Region, Inner Mongolia Agricultural University, Hohhot 010010, China; Key Laboratory of Germplasm Innovation and Utilization of Triticeae Crops at Universities of Inner Mongolia Autonomous Region, Inner Mongolia Agricultural University, Hohhot 010010, China; Key Laboratory of Germplasm Innovation and Utilization of Triticeae Crops at Universities of Inner Mongolia Autonomous Region, Inner Mongolia Agricultural University, Hohhot 010010, China; Key Laboratory of Germplasm Innovation and Utilization of Triticeae Crops at Universities of Inner Mongolia Autonomous Region, Inner Mongolia Agricultural University, Hohhot 010010, China; Beijing 8omics Gene Technology Co. Ltd, Beijing 100080, China; Beijing 8omics Gene Technology Co. Ltd, Beijing 100080, China; Inner Mongolia Guomai Agriculture Co. Ltd, Xilingol League, Xilinhot City 026005, China; Key Laboratory of Germplasm Innovation and Utilization of Triticeae Crops at Universities of Inner Mongolia Autonomous Region, Inner Mongolia Agricultural University, Hohhot 010010, China; Key Laboratory of Germplasm Innovation and Utilization of Triticeae Crops at Universities of Inner Mongolia Autonomous Region, Inner Mongolia Agricultural University, Hohhot 010010, China

**Keywords:** naked oat, genetic diversity, divergence time, introgression, selective sweep, GWAS

## Abstract

As an important cereal crop, common oat, has attracted more and more attention due to its healthy nutritional components and bioactive compounds. Here, high-depth resequencing of 115 oat accessions and closely related hexaploid species worldwide was performed. Based on genetic diversity and linkage disequilibrium analysis, it was found that hulled oat (*Avena sativa*) experienced a more severe bottleneck than naked oat (*Avena sativa var. nuda*). Combined with the divergence time of ∼51,200 years ago, the previous speculation that naked oat was a variant of hulled oat was rejected. It was found that the common segments that hulled oat introgressed to naked oat cultivars contained 444 genes, mainly enriched in photosynthetic efficiency-related pathways. Selective sweeps during environmental adaptation and breeding improvement were identified in the naked oat genome. Candidate genes associated with smut resistance and the days to maturity phenotype were also identified. Our study provides genomic resources and new insights into naked oat domestication and breeding.

## Introduction

Common oat (*Avena sativa*, NCBI:txid4498) ranks seventh in production among global cereal crops [1]. It is one of the most important crops in several countries and is widely used as human food and animal feed [2]. Oats are high in protein, are rich in polyunsaturated fatty acids, and have a low carbon footprint [3]. In recent years, oat has attracted more and more attention as healthy food due to its rich content of various bioactive compounds, which can reduce the risk of cardiovascular diseases, type 2 diabetes mellitus, gastrointestinal disorders, and cancer [4]. Oat is highly adaptable to a wide range of climates. Oat can be widely planted and have high yields in the harsh marginal environment where other major cereal crops, such as rice and corn, cannot be grown [5]. In China, naked oat (*Avena sativa var. nuda*) landraces are distributed from the warm and humid Yunnan–Guizhou region to the cold and dry Shanxi–Gansu region.

**Figure 1:**
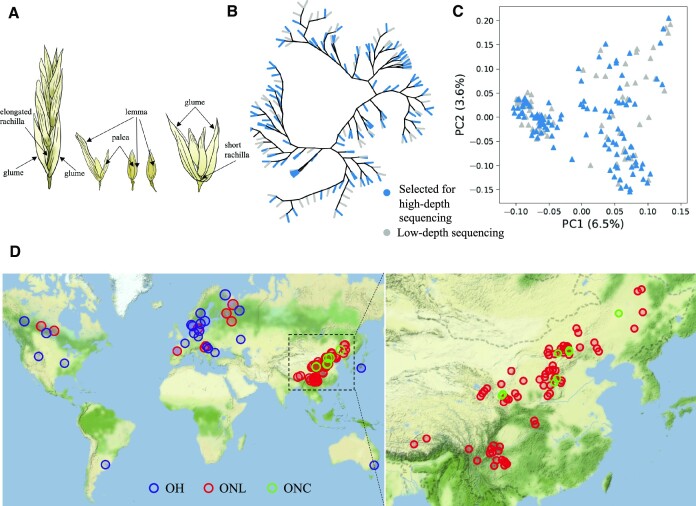
Differences between hulled and naked oat and the selection of accessions in this study. (A) Spikelet and grain of typical hulled oat (left) and naked oat (right). (B) Principal component analysis (PCA) for the 189 low-depth sequencing accessions. (C) Phylogenetic tree analysis for the 189 low-depth sequencing accessions. Blue indicates selected high-depth sequencing accessions. (D) Geographic origin of 115 selected high-depth sequencing accessions.

Common oat is typically classified into 2 types according to the morphology of the seeds: hulled and naked. Hulled oats are the widely known oats grown all over the world, while naked oat is grown mainly in China [6, 7], and the most extensive germplasm collection for naked oat is maintained at the National Germplasm Bank of China (NGBC) [8]. Hulled oat has a caryopsis tightly surrounded by a thick, lignin-rich hull that remains attached to the mature grain throughout threshing and cleaning (Fig. 1A). In contrast, naked oat is characterized by papery, free-threshing hulls that are mostly lost during threshing and cleaning [9]. Besides the free-threshing attribute, naked oat differs from hulled oat by having a multiflorous habit and elongated rachilla segments in the mature panicle [10].

Common oat is usually considered a secondary crop (i.e., derived from a weed of the primary cereal domesticates, wheat and barley) [11]. Current thinking is that common oat was probably domesticated in central or northern Europe ∼3,000 years ago from a weedy hexaploid progenitor that may have been used as a forage crop [12]. There is as yet no consensus as to the origin of naked oat. It has been proposed that the naked oat is a separate species from the hulled oat, named *Avena nuda* L. [13]; it has also been proposed that the naked oat arose as a mutant of the domesticated hulled oat. The mutant theory suggested that naked oat may have originated from hulled oat, potentially after hulled oats reached China from its main center of diversity in southwest Asia [3, 14]. However, direct molecular evidence is scarce to be reported. It has been declared the genetic diversity of naked oat is less than that of hulled oat, which would support a bottleneck effect from a mutation event to domestication [9]. However, that study was based on genotyping by sequencing, and the number of markers used was relatively low (8,675 haplotype markers).

Genome sequencing can significantly accelerate functional genomic studies of crops [15]. Owing in part to the hexaploid nature of the common oat genome (AACCDD, 2n = 6× = 42), its very large size (∼12 G), and its abundance of repetitive sequences, the genomes of hulled and naked oat have not been assembled until recently, long after other crop species such as rice, maize, and soybean. Also, there have been no large-scale resequencing studies of common oat to date. The recently published 2 high-quality genomes [3, 16] of oat will indeed trigger the climax of oat functional genome research.

Based on 455 previously defined accessions comparing the oat core collection of the NGBC [17], genetic diversity, and origin, we performed deep resequencing of 89 naked oat, 22 hulled oat, and 4 closely related hexaploid species from around the world. Our Bayesian analysis of divergence time supports that hulled oat and naked oat diverged ∼51,200 years ago, well before the domestication of oat. Moreover, the genetic diversity of naked oat is higher than that of hulled oat. Combined with other lines of evidence, we speculate that hulled oat and naked oat were domesticated independently. We also found that genetic introgression of hulled oat into naked oat cultivars increased their yield. We also investigated the genes selected during the environmental adaptation of naked oat landraces and the breeding of naked oat cultivars. Finally, we performed genome-wide association studies for 2 traits: oat smut resistance and days to maturity.

## Results

### Genetic diversity of cultivated oat

There are 3,255 oat accessions in the NGBC [8]. From among the previously defined 455 core accessions [17], 189 accessions were selected by geographic origin and subjected to low-depth whole-genome resequencing using the BGI DNBSEQ-T7 platform (average of 46.13 G bases per sample, corresponding to 3.47× coverage depth; Supplementary Table S1). The sequencing data were aligned to the OT3098 reference genome (*Avena sativa*) [16], and single-nucleotide polymorphisms (SNPs) were called using BCFtools. Based on the detected SNPs, a phylogenetic tree (Fig. 1B) and principal components analysis (PCA) (Fig. 1C) were conducted, and 115 accessions were screened by maximizing the genetic diversity and geographic origin (Fig. 1D and Supplementary Table S2) for deep sequencing (average sequencing depth 13.17×; Supplementary Table S1), including 89 naked oats (81 landraces [“ONL”], 8 cultivars [“ONC”]), 22 hulled oats (“OH”), and 4 closely related hexaploid species (*Avena fatua* L., *Avena occidentalis* Dur., *Avena sterilis* L,. and *Avena byzantina* Koch.).

**Figure 2:**
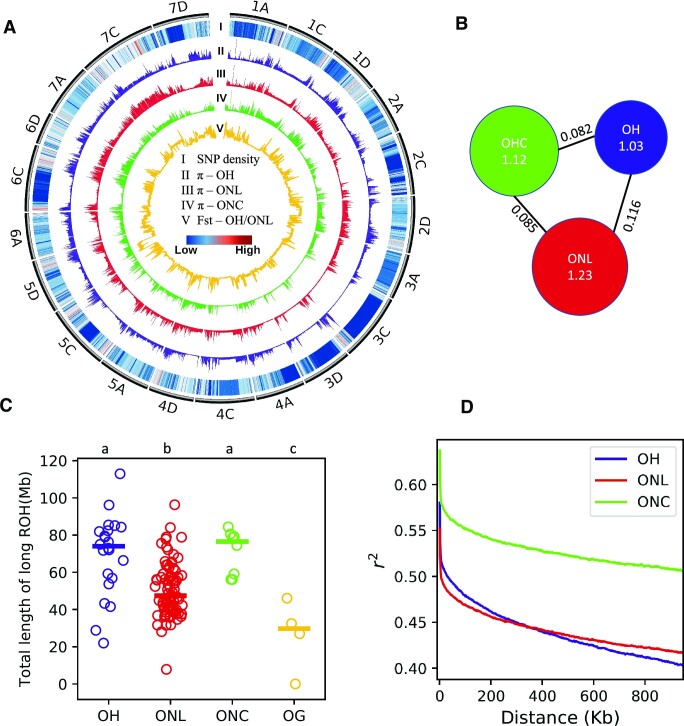
Genetic diversity of common oat. (A) The distribution of SNP density, genetic diversity, and distance across chromosomes. The outer gray tracks represent the chromosomes of the OT3098 reference genome. (B) The genetic diversity (π) and distance (Fst) of OH, ONL, and ONC. (C) The runs of homozygosity (ROH) of OH, ONL, ONC, and OG. (D) The linkage disequilibrium (LD) decay analysis for OH, ONL, and ONC.

The average Q20 and Q30 ratios of sequenced data were 97.92% and 93.29%, respectively (Supplementary Table S2), indicating the very high quality of the data. The average mapping rate was 99.30%, and the average coverage rate was 94.96% (Supplementary Table S1). A total of 336,725,135 SNPs were detected in the 115 accessions. SNPs with low quality and minor allele frequency (MAF) <0.05 and missing rate >20% were filtered out, leaving 52,817,822 SNPs for subsequent analyses (Fig. 2A). The transitions versus transversions ratio of the SNPs was 1.93, which was very close to the expected value of 2, indicating the high quality of the SNP set.

Calculating the nucleic acid diversity (π) and genetic distance (Fst) of the ONL, ONC, and OH sets of accessions (Fig. 2A,B) showed that the extent of polymorphism was highest in the ONL accessions (π = 1.23e^−3^), followed by ONC (π = 1.12e^−3^) and then OH (π = 1.03e^−3^). The genetic distance between ONL and OH is the farthest (Fst = 0.116), and the genetic distance between ONC and ONL/OH is similar (Fst = 0.085 vs. Fst = 0.082). This may be related to the breeding history of hulled–naked hybridization in ONC [18].

The runs of homozygosity (ROH) analysis was conducted, which indicated the following order for the degree of inbreeding (highest first): ONC, OH, ONL, and the closely related hexaploid species (OG) (Fig. 2C). We detected strong linkage disequilibrium (LD) in the oat genome (Fig. 2D) and noted that the *r*^2^ of LD decay was greater than 0.4 for windows larger than 1 Mb in all populations. This level of LD was much higher than in other crops such as rice [19], maize [20, 21], and sorghum [22, 23]. Because cross-breeding would lead to increased LD [24], the LD of ONC was much higher than that of OH and ONL.

### Differentiation of hulled oat and naked oat

**Figure 3:**
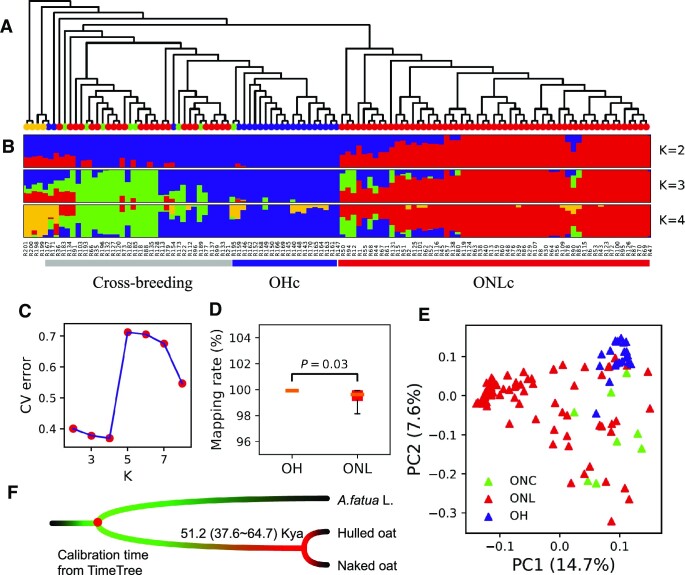
Population structure and differentiation of hulled oat and naked oat. (A) Neighbor-joining (NJ) tree based on identity by state (IBS) distance. (B) A model-based ancestry estimating conducted using ADMIXTURE. (C) Cross-validation error when k = 2 to 8. (D) Reads mapping rates of OH and ONL. (E) PCA plot of the first 2 principal components for all accessions. Closely related hexaploid species were excluded in this analysis. (F) Divergence time of hulled oat and naked oat estimated using BEAST. The divergence time (∼0.78 million years ago) of *A. fatua* L. and cultivated oat was used as calibration.

Although early studies proposed that naked oat was a separate species [13] (*A. nuda* L.), researchers now generally regard naked oat as a variant of hulled oat, potentially resulting from a mutation that occurred after hulled oat was introduced into China [3, 9]. A phylogenetic tree clustered hulled oat and naked oat landraces as 2 independent clades (Fig. 3A). We named these 2 separate clades OHc (*n* = 19) and ONLc (*n* = 57), respectively. Some accessions did not cluster according to the hulled–naked phenotype, reflecting cross-breeding. ADMIXTURE analysis (Fig. 3B) found that the cross-validation error rate was the smallest when K = 4 (Fig. 3C). At this level, the accessions were clustered as ONLc, OHc, cross-breeding group, and OG. The cross-breeding group included all ONC and 15 ONL, indicating that naked oat landraces were also improved by crossing with hulled oat. Seven of the 9 ONL collected from outside China were clustered in the cross-breeding group.

It was mentioned above that the genetic diversity of ONL was higher than that of OH. To avoid the effects of gene flow and sample size, 100 random samplings (*n* = 19, consistent with the sample size of OHc) of ONLc were performed to calculate genetic diversity. We found that the genetic diversity of ONLc was still higher than that of OHc (1.08e^−3^ vs. 0.90e^−3^). Although the mapping rates of ONL and OH were high and not significantly different (99.04% vs. 99.92%; Fig. 3D), the mapping rates of ONL had greater variation (standard deviation: 1.91 vs. 0.02). The PCA results showed that ONL occupied the largest variable space (Fig. 3E), consistent with the genetic diversity finds. These contradicted the speculation that the naked oat is a variant of the hulled oat, in which the naked oat would experience an additional bottleneck, resulting in lower genetic diversity than the hulled oat. Moreover, the hulled oat had a stronger short-distance LD (Fig. 2D), indicating that the hulled oat experienced a more severe bottleneck than the naked oat. Combining hulled oat and naked oat had a deep split on the phylogenetic tree, these lines of evidence argue against the idea that naked oats originated as a variant of hulled oats.

The differentiation time of hulled oat and naked oat was also modeled. To calculate the divergence time between hulled oat and naked oat, all high-quality fourfold degenerate loci loci genotypes in the whole genome were extracted. Through these neutral evolutionary sequences and using a calibration time (∼0.78 million years ago) based on the closely related hexaploid species *A. fatua* and cultivated oats on TIMETREE, the differentiation time of hulled oat and naked oat was predicted as ∼51,200 years ago (Fig. 3F). We have also separately calculated the divergence times of the 3 subgenomes. The divergence time of subgenome A was ∼47.3 Kya (thousand years ago), subgenome C was ∼47.0 Kya, and subgenome D was ∼53.3 Kya. This time predates the domestication of wheat ∼10,000 years ago [25, 26]. Cultivated oat was domesticated as a field weed of wheat [11], indicating that hulled oat and naked oat had diverged before domestication and underwent an independent domestication process.

### Introgression from hulled oat into naked oat cultivars

**Figure 4:**
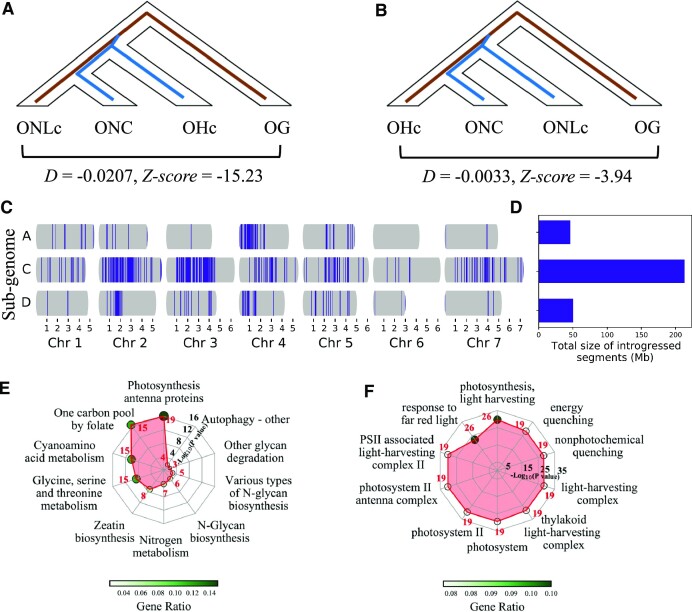
Introgression analyses between OH and ONC. (A) Patterson's D statistic for testing introgression between ONC and OHc. Brown lines represent shared derived alleles, while blue lines represent shared more derived alleles than expected due to introgression. (B) Patterson's D statistic for testing introgression between ONC and ONLc. (C) The common introgressed segments (blue) across chomosomes revealed by rIBD analysis. (D) Barplot of the total length of introgressed segments from different subgenomes. (E, F) The GO and KEGG enrichment of the 444 introgressed genes. The plots shows the top 10 most significantly enriched terms or pathways.

The breeding of naked oat in China has gone through phases, including the direct collection and utilization of landraces, cross-breeding between naked oat varieties, and cross-breeding of naked oat with hulled oat [18]. In particular, the cross-breeding between hulled oat and naked oat is recognized to have increased the yield of naked oat by more than 30% [18]. The national average yield increased from 75 kg/mu in 1998 to 150 kg/mu in 2014 [27]. Consistently, the ADMIXTURE and phylogenetic tree analyses supported the cross-breeding history of ONC. In addition, Patterson's D statistical analysis (Fig. 4A) indicated that ONC and OHc accessions share more derived alleles than would be expected by chance (D = −0.0207, *z*-score = −15.23). Patterson's D statistical analyses even indicated that ONC and OHc shared more derived alleles than ONLc (D = −0.0033 vs. D = −0.0207; Fig. 4A,B).

To investigate the common introgression segments that OHc has contributed to ONC, the relative identical by descent (rIBD) analysis was performed [28, 29]. This predicted the total length of the OHc segments introgressed into ONC as 309 Mb (2.8% of the whole genome) (Fig. 4C). Most (76.6%) of these introgressed segments are on chromosomes 2C, 3C, 4A, 4C, 4D, 5C, and 7C and contain a total of 444 predicted open reading frames (ORFs) (Supplementary Table S3). Note that the total length of introgressed segments in subgenome C was 4.63 and 4.24 times that of subgenomes A and D, respectively (Fig. 4D). Exploratory analyses using GO and KEGG indicated that this set of introgressed genes is enriched for annotations related to photosynthesis (Fig. 4E,F and Supplementary Tables S4 and S5), suggesting that hulled oat may have contributed to yield improvements for naked oat cultivars by altering photosynthetic efficiency.

Among these 444 introgressed genes, many predicted gene products could be possibly related to differential yield, including, for example, a VIN3-like protein (Pepsico2_Contig10032), ABC transporters (Pepsico2_Contig7110, Pepsico1_Contig27635, Pepsico2_Contig16075, Pepsico1_Contig16853), a polypyrimidine tract-binding protein homolog 1-like (Pepsico2_Contig20359), a BTB/POZ domain-containing protein (Pepsico2_Contig17276), cytochrome P450 enzymes (Supplementary Table S6), a GDSL esterase/lipase (Pepsico2_Contig5911), bZIP transcription factors (Supplementary Table S6), and light-harvesting chlorophyll a/b binding proteins (Supplementary Table S6).

### Environmental adaptability of naked oat landraces and artificial selection of cultivars

Oat is highly adaptable to various climates, including arid and cold regions, and is an excellent species for studying crop abiotic stress tolerance [30, 31]. Studying the environmental adaptability of oat can point toward genetic mechanisms and can also provide guidance for the genetic improvement of oat. To study the environmental adaptation mechanisms of naked oats, we used 2 clades of ONL on the phylogenetic tree: one for accessions from arid and low-temperature regions of China, including Gansu, Ningxia, and Qinghai (group GNQ), and one for accessions from the Yunnan, Sichuan, and Guizhou (group YG), 3 provinces with relatively higher rainfall and temperature (Supplementary Table S7).

**Figure 5:**
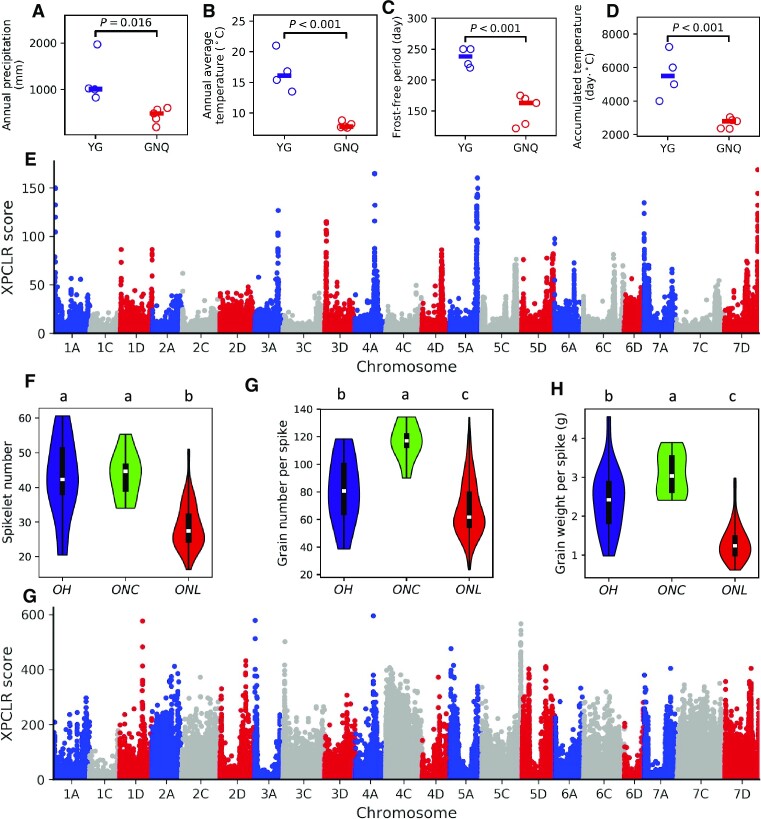
The selective sweep analyses for local environment adaptation of ONL and improvement of ONC. (A–D) The annual precipitation, annual average temperature, frost-free period, and accumulated temperature of YG and GNQ. YG represented accessions from provinces with relatively higher rainfall and temperature, and GNQ represented accessions from arid and low temperature regions of China. (E) Selection signatures in GNQ genomes while using YG as reference. (F–H) The spikelet number, grain number per spike, and grain weight per spike of OH, ONC, and ONL. Lowercase letters on the top of the plots indicate significant differences among groups (*P* < 0.01, Student's *t*-test). (G) Selection signatures in ONC genomes while using ONLc as reference.

The annual rainfall, average temperature, frost-free period, and accumulated temperature of YG were significantly higher than that of GNQ by Student's *t*-test: the annual rainfall (1,203.75 mm vs. 440.00 mm, *P* = 0.016; Fig. 5A), the annual average temperature (16.67°C vs. 8.02°C, *P* = 0.00049; Fig. 5B), frost-free period (236.5 days vs. 151.82 days; Fig. 5C), and accumulated temperature (5,556.0°C vs. 2,684.4°C, *P* = 0.0025; Fig. 5D). A selective sweep analysis was performed that predicted 8,620 selective sweep signatures in the group GNQ when using the group YG as a reference (Fig. 5E). Notably, a gene enrichment analysis indicated that the genes within the selective sweep regions were enriched for functional annotations related to drought resistance, cold acclimation, and DNA damage repair (Supplementary Tables S8 and S9).

Naked oat breeding programs have successfully improved cultivars’ yield and lodging resistance [18, 27]. We investigated yield-related agronomic traits, including spikelet number, grain number per spike, spikelet length, panicle length, and grain weight per spike for OH, ONL, and ONC (Supplementary Table S10). It was found that ONC was significantly improved over ONL among all these phenotypes (*P* < 0.01, Student's *t*-test). In particular, spikelet number (Fig. 5F), grain number per spike (Fig. 5G), and grain weight per spike (Fig. 5H) were greatly improved.

Seeking to identify genes that were selected during the improvement of ONC, we performed a selective sweep analysis using ONLc as a reference (Fig. 5G). A total of 7,667 selective sweep signatures were predicted. The set of genes within the putatively selected regions was enriched for annotations, including “carbohydrate mediated signaling,” “sugar mediated signaling pathway,” and “regulation of developmental vegetative growth” (Supplementary Tables S11 and S12). Some of the selected genes may be related to cultivars’ improved yield and lodging resistance. For example, previous studies have reported that cellulose synthase enzymes can improve the lodging resistance of oat stems by increasing the content of the stem structural carbohydrate cellulose [32, 33]. There were 30 genes related to cellulose synthesis among the genes from the candidate selective sweep regions (Supplementary Table S13). Many yield-related genes were among the candidate regions, such as *AP2* (13 genes were annotated as *AP2*; Supplementary Table S12). *APETALA 2* (*AP2*)–like family plays an essential role in inflorescence and spikelet development [34]. These candidate genes can provide helpful information for future oat improvement.

### Association analysis between smut resistance of oat and days to maturity

**Figure 6:**
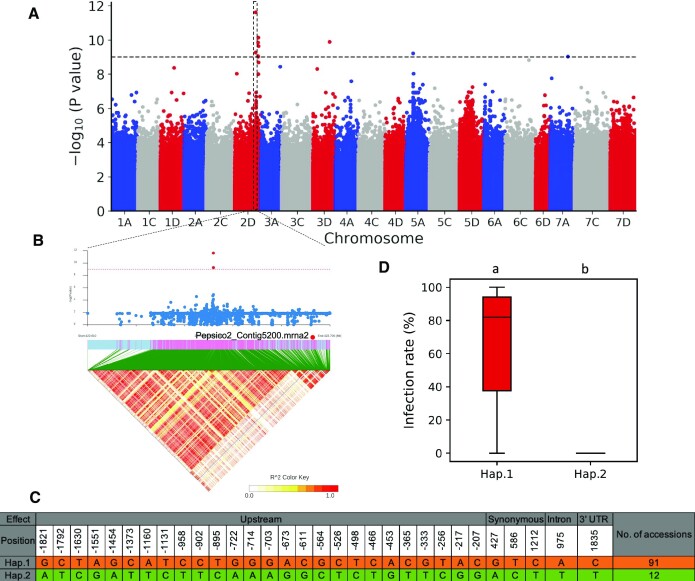
Genetic loci associated with the smut resistance. (A) Manhattan plot for the GWAS result. The horizontal dotted line depicts the Bonferroni-adjusted significance threshold (α = 0.05). (B) The local association signature and LD block heatmap. The red dots represent significantly associated SNPs. (C) The haplotypes constructed using SNPs in the candidate gene or in 2 kb upstream of the candidate gene. (D) The phenotypes of different haplotypes. Lowercase letters on the top of the plot indicate significant differences between Hap.1 and Hap.2 (*P* < 0.01, Student's *t*-test).

Oat smut is a major oat disease caused by fungal pathogens of the family Ustilaginaceae [35]; it affects the heading stage and seriously reduces oat yields [36]. To enable an association analysis, phenotypic data of the 115 accessions (Supplementary Table S2) were downloaded from the Chinese Crop Germplasm Resources Information System (CCGRIS) [8]. The phenotype data were collected in experiments that used uniform strains and inoculation methods, and 3-year averages were taken. Using the detected 52,817,822 SNPs, we performed a genome-wide association study (GWAS) and identified a significant association signal on oat chromosome 2D (Fig. 6A). Five predicted ORFs contained significant SNPs or were located upstream and downstream of significant SNPs. A gene (Pepsico2_Contig5200) positioned 43 kb from the most significant SNP was annotated as Zealexin A1 synthase (Fig. 6B). In maize, CYP71Z18 catalyzes the formation of maize phytoalexins, including zealexin A1. Overexpression of CYP71Z18 in rice resulted in the accumulation of several new diterpenoids, and transgenic rice also showed stronger resistance to rice blast infection [37]. There are 31 SNPs within the gene and a 2-kb upstream region, of which 3 SNPs are synonymous mutations: one is located in an intron, and one is located in the 3′-UTR. The 31 SNPs constituted 2 haplotypes, with Hap.1 containing 91 accessions and Hap.2 containing 12 accessions (Fig. 6C). All accessions carrying Hap.1 were susceptible to smut infection, while all accessions carrying Hap.2 had some degree of smut resistance (Fig. 6D). Hap.1 can be used as a reliable molecular marker for marker-assisted selection (MAS) to develop new smut-resistant varieties.

**Figure 7:**
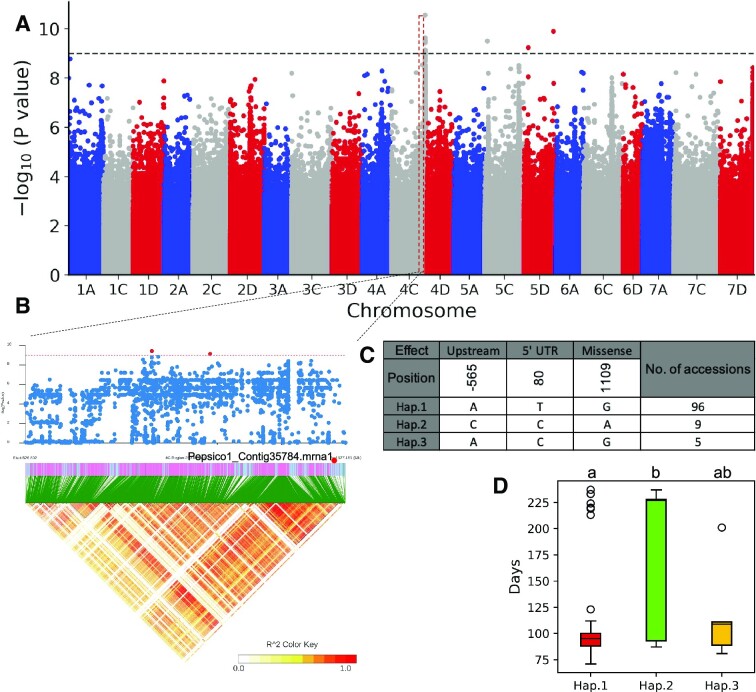
Genetic loci associated with the DTM. (A) Manhattan plot for the GWAS result. The horizontal dotted line depicst the Bonferroni-adjusted significance threshold (α = 0.05). (B) The local association signature and LD block heatmap. The red dots represent significantly associated SNPs. (C) The haplotypes constructed using SNPs in the candidate gene or in 2 kb upstream of the candidate gene. (D) The phenotypes of different haplotypes. Lowercase letters on the top of the plot indicate significant differences among haplotypes (*P* < 0.01, Student's *t*-test).

An agronomic trait known as days to maturity (DTM) describes the average number of days from planting until harvest. For a given species, in general, the longer the DTM, the higher the yield [38]. To obtain candidate genes associated with DTM, GWAS was performed using the SNPs mentioned above and phenotypic data for the 115 accessions downloaded from the CCGRIS. These phenotypic data were the mean of 3-year measurements in origin or adapted ecoregion (i.e., under normal growth conditions). Association analysis found a significant association signal on oat chromosome 4C (Fig. 7A). Eleven predicted ORFs contained significant SNPs or were located upstream and downstream of significant SNPs. Among them, Pepsico1_Contig35784.mrna1 has been annotated as a pentatricopeptide repeat-containing protein (Fig. 7B). A previous study showed that loss of function of this gene promoted early flowering in Arabidopsis [39]. There are 3 SNPs in the gene: 1 located 2 kb upstream of the gene, 1 located in the 5′-UTR region, and another in an exon that is predicted to cause a missense mutation. These 3 SNPs constitute 3 haplotypes (Fig. 7C). Ninety-six accessions were carrying Hap.1 with an average DTM of 103.6 days, 9 accessions were carrying Hap.2 with an average DTM of 182.7 days, and 5 accessions were carrying Hap.3 with an average DTM of 118.2 days (Fig. 7D).

## Discussion

A total of 115 oat accessions worldwide, including 89 naked oats (81 landraces and 8 cultivars), 22 hulled oats, and 4 closely related hexaploid species, were collected. The genetic diversity of each oat population was calculated based on high-depth sequencing data for these 115 accessions. It was found that the genetic diversity of naked oat was higher than that of hulled oat (1.23e^−3^ vs. 1.12e^−3^). This is contrary to previous reports [3, 9] and could reflect the low number of markers used in those studies (8,675 haplotype markers). We found considerable differences in genetic diversity in different regions of chromosomes, such as ONLc, and the mean π of chromosomes with the largest and smallest genetic diversity were 0.47e^−3^ and 2.45e^−3^ (Fig. 2A), respectively. Therefore, a small number of markers may lead to sampling bias.

Moreover, compared with naked oat, hulled oat had stronger short-distance linkage disequilibrium. These pieces of evidence suggested that hulled oat experienced a more severe bottleneck than naked oat, which contradicts the previously reported speculation that naked oat originated as a variant of hulled oat [9]. If naked oat is a variant of hulled oat, naked oat would experience an additional bottleneck after the domestication bottleneck shared with hulled oat. Furthermore, PSMC′ (a special case of MSMC for 2 haplotypes) was used to calculate the demographic history. We found that regardless of whether all chromosomes or 3 subsets of subgenomes were calculated separately, the effective population size changes of hulled and naked oat were very similar: they have been decreasing since 1 million years ago until now. Compared with hulled oat, naked oat did not experience a more severe bottleneck (Supplementary Fig. S1). By calculating the divergence time of hulled oat and naked oat, we estimate that these 2 oat types differentiated ∼51,200 years ago, much earlier than the estimated domestication time of ∼3,000 years ago for oat [12]. Distinct from multiple previous proposals [3, 9], these lines of evidence from our study suggest that hulled oat and naked oat were domesticated independently.

Crop domestication and improvement history, however, is a complex research topic. Like other crops such as rice [40–44], maize [45–49], and wheat [50–53], it often requires more genomic data as well as archeological and geographical evidence. Therefore, the conclusions drawn from our article based on existing data may have certain limitations. For instance, the lack of more genomic data from closely related hexaploid oats limits our understanding of gene flow in domestication and improvement [54]. More research is needed to further reveal the clear outline of oat domestication and improvement history.

Through the efforts of breeders in recent decades, the yield of naked oat has been dramatically improved [18, 27]. The way to achieve this includes a continuous artificial selection of landraces and cross-breeding with hulled oat. We found genetic evidence for both ways. Through selective sweep analysis of ONC, we identified a large number of selective signatures of genes related to yield and lodging resistance. The cross-breeding history has also been revealed by Patterson's D statistic and rIBD analyses. The common introgressed segments from hulled oat in OHC contained 444 genes enriched for annotations related to photosynthesis. Compared with ONL, yield-related traits of ONC mainly improved spikelet number, grain number per spike, and grain weight per spike. In addition, our GWAS results provided MAS markers for future genetic improvement of oat.

There is a large amount of arid and semiarid land worldwide [55]. Research on the genetic mechanism of drought resistance of crops can help to develop new varieties of drought-resistant crops and promote human food security [56, 57]. Oat is often grown in harsh, semiarid environments, making them an excellent species to study the genetic mechanisms of drought resistance [5]. Candidate genes associated with drought resistance were obtained through selective sweep analysis using naked oat landraces from arid environments. For example, among the candidate genes, there were 43 UDP-glucosyltransferases. A previous study reported that overexpression of UDP-glycosyltransferase 3 (*UGT3*) can enhance rice's drought and salt stress tolerance [58]. In Arabidopsis, overexpression of *UGT79B2/B3* significantly enhanced plant tolerance to low temperature as well as drought and salt stress, whereas *ugt79b2/b3* double mutants generated by RNA interference and CRISPR-Cas9 were more susceptible to adverse environmental conditions [59]. These candidate genes can provide helpful information for future studies on oat drought resistance.

In summary, our study provides valuable genomic resources for oat genomic and genetic research. We have overturned proposals about the origin of the naked oat and raised the idea that the naked oat was independently domesticated. Through introgression, selective sweep, and GWAS analyses, we provide a genomic framework and valuable information for facilitating marker-assisted selection for oat breeding.

## Materials and Methods

### Plant materials

The seeds of the 189 selected accessions mentioned above were sowed in a seedling tray at a depth of 2 cm, keeping the soil moist. When the oats grew to the 3-leaf stage, we collected leaves from 3 plants for each accession, mixed the samples, placed them in 2-mL centrifuge tubes, and immediately stored them in liquid nitrogen. The sample source map was visualized using the Python package folium. The background map used is from Stamen Design [60].

### Whole-genome resequencing

Genomic DNA was extracted from young leaves using DNeasy Plant Mini Kits (Qiagen GmbH). The quality and concentration of DNA were assessed by 1.0% agarose gel electrophoresis and using a nanodrop spectrophotometer (Thermo Fisher Scientific). Sequencing library construction was performed using the MGIEasy FS PCR-Free DNA Library Prep Set (MGI Tech), following the manufacturer's instructions. DNA sequencing was performed using the 2 × 150-bp paired-end mode of the DNBSEQ-T7 platform (MGI Tech; RRID:SCR_017981).

### SNP calling

The quality of the generated sequencing data was assessed using fastp (RRID:SCR_016962) [61], and high-quality reads were aligned to the OT3098 reference genome [62] using BWA mem (RRID:SCR_010910) [63] with the default parameters. SAMtools fixmate (RRID:SCR_002105) [64] was used to fill in the mate coordinates, insert sizes, and mate-related flags. Polymerase chain reaction duplicates were marked using SAMtools markdup. BCFtools (RRID:SCR_005227) [65] was used to perform joint SNP calling. To reduce false positives, genotypes with quality less than 10 were marked as missing, and SNPs with “QUAL < 30 | INFO/FS > 60.0 | INFO/MQ < 40.0 | F_PASS(GQ> = 10 & GT! = “mis”) < 0.8 | MAF < 0.05” were filtered out. Gene-based SNP annotation was performed using the SnpEff (RRID:SCR_005191) [66] software package.

### Phylogenetic tree and population structure analyses

An identity by state (IBS) distance-based neighbor-joining tree was built using the bionj function of the R package ape (RRID:SCR_017343) [67]. The pairwise IBS distances of all oat accessions were estimated using PLINK (RRID:SCR_001757) [68]. A model-based ancestry estimation was conducted using the program ADMIXTURE (RRID:SCR_001263) [69] with K = 2 to 8. The cross-validation procedure was used to determine the K number of the best-fit model. PCA was performed using the smartPCA program implemented in the EIGENSOFT (RRID:SCR_004965) [70] package.

### Genetic diversity and genetic distance analyses

Nucleotide diversity (θπ, the average number of pairwise nucleotide differences per site between any 2 randomly chosen DNA sequences from the population) and fixation index (Fst) across the whole genome were calculated using VCFtools (RRID:SCR_001235) [71] with a sliding window of 1 Mb and a step size of 500 kb. ROH segments were detected using PLINK (RRID:SCR_001757) [68] with the parameters “-homozyg-window-snp 50 –homozyg-window-missing 2 –homozyg-window-het 0 –homozyg-snp 50 –homozyg-kb 500 –homozyg-density 50.”

### Linkage disequilibrium decay

To estimate and compare LD decay patterns, we used PopLDdecay (RRID:SCR_022509) [72] to calculate the mean squared correlation coefficient (*r*^2^) values of all SNP pairs within 1 Mb. A bin size of 500 bp was used to generate the LD decay plot.

### Divergence time estimation

To estimate the divergence time between hulled oat and naked oat, we identified all 6,814,874 fourfold degenerate loci according to the gene models of the oat reference genome gene annotations. These loci from 3 high-depth sequencing accessions (R86 from naked oat, R148 from hulled oat, and *A. fatua*) were then genotyped using BCFtools (RRID:SCR_005227) [65]. To obtain high-confidence genotypes, loci with QUAL less than 60 or GQ less than 30 in any accession were filtered out. Finally, 38,028 loci were left to estimate the divergence time. The divergence time was estimated using an uncorrelated relaxed clock in BEAST (RRID:SCR_010228) [73]. The divergence time between *A. fatua* and *A. sativa* acquired from TIMETREE (RRID:SCR_021162) [74] was used to calibrate the evolutionary rate (Blosum62 and an uncorrelated exponential relaxed model). The Yule speciation process was used, which specifies a constant rate of species divergence. Normal priors were used for hulled oat and naked oat divergence time. The chain length was set to 10,000,000, sampling every 1,000 steps. Tracer (RRID:SCR_019121) [75] was used for visualizing and analyzing the Bayesian markov chain monte carlo (MCMC) runs.

### Demographic history

To infer demographic history, MSMC2 [76] was used. To ensure reliability, 9 accessions (R57, R60, R67, R70, and R86 for naked oat and R148, R164, R169, and R170 for hulled oat) without admixture between naked oat and hulled oat (indicated by ADMIXTURE and TREE) were selected for analysis with a sequencing depth of more than 15×. Genome regions were masked when the coverage depth was below 15× after removing reads with mapping quality <20 and were also masked using Heng Li's SNPable tool [77]. In brief, the reference genome was split into overlapping 35-mers and then mapped back to the reference genome using BWA (bwa aln -R 1,000,000 -O 3 -E 3). Only regions of the majority of overlapping 35-mers mapped back uniquely and without 1-mismatch were kept. Scaled times were converted to years assuming a generation time of 1 year and a mutation rate of 6.5 × 10^–9^ per site per generation. The atmospheric surface air temperature relative to the present (°C) and the ice volume contribution to the marine isotope signal (relative to the present) were downloaded from the national climatic data center (NCDC) database [78].

### Introgression analysis

To detect introgression between naked oat cultivars and hulled oat, we used Patterson's D statistic [79], which was implemented in the R package admixtools (RRID:SCR_018495) [80], to test gene flow with 4 hexaploid relatives as the outgroup. The significance was assessed by a block jackknife procedure. To clarify the genomic location of the candidate introgression segments, we performed rIBD [81] analysis. First, we phased the genotypes using Beagle (RRID:SCR_001789) [82] and then used RefinedIBD [83] to detect shared IBD tracks between any 2 accessions. We counted the number of shared IBD tracks with a 100-kb sliding window and a 50-kb step in comparing each naked oat cultivar with ONL or OH accessions. These counts were then normalized as nIBD = shared IBD number/number of ONL or OH, and the rIBD was calculated as rIBD = nIBD_OH_ − nIBD_ONL_. rIBD values of all windows were calculated and then normalized following a standard normal distribution. Windows with *z*-scores greater than 2 were considered putative introgression segments.

### Gene enrichment analysis

GO and KEGG enrichment analyses of selected genes were performed using the R package ClusterProfiler (RRID:SCR_016884) [84]. The terms with an adjusted *P* value less than 0.05 were considered significantly enriched.

### Selective sweep analysis

Selective sweep analysis was performed using a reimplementation of the Python version [85] of XPCLR [86], with a window size of 100 kb and a step size of 20 kb. Other parameters were set as –rrate 1e-8 –ld 0.95 –phased –maxsnps 200 –minsnps 10. The windows with the top 5% XPCLR scores were considered putative selective sweep regions.

### Whole-genome association study

A linear mixed model implemented in the GEMMA [87] software toolkit was used for the GWAS. Bonferroni correction was used to control the false discovery rate for multiple testing, with a significant level of 0.05 (α = 0.05). Linkage disequilibrium blocks were detected and visualized using PopLDdecay [72].

## Supplementary Material

giad061_GIGA-D-22-00306_Original_Submission

giad061_GIGA-D-22-00306_Revision_1

giad061_GIGA-D-22-00306_Revision_2

giad061_Response_to_Reviewer_Comments_Original_Submission

giad061_Response_to_Reviewer_Comments_Revision_1

giad061_Reviewer_1_Report_Original_Submission Mona Schreiber -- 2/23/2023 Reviewed

giad061_Reviewer_1_Report_Revision_1 Mona Schreiber -- 5/21/2023 Reviewed

giad061_Reviewer_2_Report_Original_Submission Xupo Ding -- 2/6/2023 Reviewed

giad061_Reviewer_2_Report_Revision_1 Xupo Ding -- 5/28/2023 Reviewed

giad061_Reviewer_2_Report_Revision_2 Xupo Ding -- 6/13/2023 Reviewed

giad061_Supplemental_Table

## Data Availability

The raw sequence data reported in this article have been deposited into the China National GeneBank DataBase under accession number CNP0003840, and the Sequence Read Archive of the National Center for Biotechnology Information under accession number PRJNA921897. All supporting data and materials are available in the *GigaScience* GigaDB database [88].

## Additional files

**Supplementary Table S1**. Mapping summary of the 189 accessions.

**Supplementary Table S2**. General information of the selected 115 accessions for high-depth sequencing.

**Supplementary Table S3**. The gene annotations for the 444 introgressed genes.

**Supplementary Table S4**. The GO enrichment analysis for the introgressed genes.

**Supplementary Table S5**. The KEGG enrichment analysis for the introgressed genes.

**Supplementary Table S6**. Genes possibly related to differential yield in the 444 introgressed genes.

**Supplementary Table S7**. Climatic data of selected accessions for selective sweep analysis.

**Supplementary Table S8**. The GO enrichment analysis result for arid adaptation.

**Supplementary Table S9**. The KEGG enrichment analysis result for arid adaptation.

**Supplementary Table S10**. Yield-related phenotypic data.

**Supplementary Table S11**. The GO enrichment analysis result for OHC improvement.

**Supplementary Table S12**. The KEGG enrichment analysis result for OHC improvement.

**Supplementary Table S13**. The candidate gene annotations for ONC improvement.

**Supplementary Fig. S1**. Demographic history inferred using MSMC2. Nine accessions (R57, R60, R67, R70, and R86 for naked oat and R148, R164, R169, and R170 for hulled oat) without admixture between naked oat and hulled oat (indicated by ADMIXTURE and TREE) were selected for analysis. The ice volume and global surface air temperature, as reconstructed using benthic oxygen isotope stack, are shown as gray solid and dotted lines, respectively.

## Abbreviations

bp: base pair; CCGRIS: Chinese Crop Germplasm Resources Information System; DTM: days to maturity; GO: Gene Ontology; GWAS: whole-genome association study; IBS: identity by state; kb: kilobase; KEGG: Kyoto Encyclopedia of Genes and Genomes; LD: linkage disequilibrium; MAS: marker assisted selection; Mb: megabase; NGBC: National Germplasm Bank of China; ORF: open reading frame; PCA: principal components analysis; rIBD: relative identical by descent; ROH: runs of homozygosity; SNP: single-nucleotide polymorphism; UTR: untranslated region.
