# Supplementary material for: Genome resequencing reveals independent domestication and breeding improvement of naked oat
Source: Gigascience. 2023 Aug 1;12:giad061. doi: 10.1093/gigascience/giad061 (PMC10390318; doi:10.1093/gigascience/giad061)

GigaScience

Genome resequencing reveals independent domestication and breeding improvement of naked oat

--Manuscript Draft--

**Manuscript Number:**	GIGA-D-22-00306R1
**Full Title:**	Genome resequencing reveals independent domestication and breeding improvement of naked oat
**Article Type:**	Research
**Funding Information:**	National Key Research and Development Project of China (2022YFE0119800) — Dr. Bing Han<br>Funding for Key Laboratory of Inner Mongolia Autonomous Region — Dr. Bing Han<br>Funds for Educational Development and Reform Platform of Inner Mongolia Autonomous Region (2100001184) — Dr. Bing Han
**Abstract:**	As an important cereal crop, common oat has attracted more and more attention due to its healthy nutritional components and bioactive compounds. Here, high-depth resequencing of 115 oat accessions and closely related hexaploid species worldwide was performed. Based on genetic diversity and linkage disequilibrium analysis, it was found that hulled oat (Avena sativa) experienced a more severe bottleneck than naked oat (Avena sativa var. nuda). Combined with the divergence time of ~51,200 years ago, the previous speculation that naked oat was a variant of hulled oat was rejected. It was found that the common segments that hulled oat introgressed to naked oat cultivars contained 444 genes, mainly enriched in photosynthetic efficiency-related pathways. Selective sweeps during environmental adaptation and breeding improvement were identified in the naked oat genome. Candidate genes associated with smut resistance and the days to maturity phenotype were also identified. Our study provides genomic resources and new insights into naked oat domestication and breeding.
**Corresponding Author:**	Bing HAN, Ph.D<br>Inner Mongolia Agriculture University: Inner Mongolia Agricultural University<br>hohhot, Inner Mongolia CHINA
**Corresponding Author Secondary Information:**	
**Corresponding Author's Institution:**	Inner Mongolia Agriculture University: Inner Mongolia Agricultural University
**Corresponding Author's Secondary Institution:**	
**First Author:**	Bing Han
**First Author Secondary Information:**	
**Order of Authors:**	Bing Han<br>Jinsheng Nan<br>Yu Ling<br>Jianghong An<br>Ting Wang<br>Mingna Chai<br>Jun Fu<br>Gaochao Wang<br>Cai Yang

Powered by Editorial Manager® and ProduXion Manager® from Aries Systems Corporation

	Yan Yang
**Order of Authors Secondary Information:**	
**Response to Reviewers:**	Dear Reviewers and Editors,<br>We appreciate your help in reviewing our work very much. All of the valuable comments and suggestions have helped improve the quality of our manuscript. Below, we have responded to the reviewers' comments point-by-point, and all corresponding improvements have been tracked and highlighted within the manuscript. Considering each author's contributions after the revised version, we have modified the order of the authors with the agreement of all authors involved. Additionally, we have adjusted the funding information supporting this work following the requirements of the funding organization.<br>If you have more questions, please contact us at hb_nmg@163.com<br>Best regards<br>Han Bing<br><br><br>Reviewer reports:<br><br>Reviewer #1: Dear Authors,<br><br>It was very fascinating to read your new genomic insights into the domestication histories of naked and hulled oats. The discovery of such a long divergence period between naked and hulled oats as well as the suggested genomic differences would add a whole new flavor to the domestication history of oats.<br><br>Looking into the data makes it clearly an interesting resource that will provide a new foundation for further analyses.<br>Unfortunately, you haven't ruled out any demographic effects, which lets me question your results and thus its interpretation.<br><br>Regarding the very distinct origin of your samples and the heterogenous sample sizes, demographic analysis would add robustness to your analyses, especially to get a clearer insight into the presumably secondary domestication history. We assume that the domestication history of oat is a complex one with a secondary origin from a weed. So imagine, that the weedy oat (which was already adapted to some part to the human made agricultural setting in its area of origin - SW Asia? Mediterranean?), was transported to different regions (as e.g. Eastern Asia and Central Europe) and only then followed by the final steps towards domestication? Hexaploid oats are to my knowledge fully inter-fertile and therefore cross-pollination can easily increase heterogeneity and within group diversity.<br><br>As we do assume that oat experienced a more complex domestication history I am very much looking forward to see data resources, such as what you provide here, will help to shed light into such complex histories. Nevertheless, I also think that we need to be very careful when analysing genomic data to illuminate our understanding of cultivation histories that are known to be not as straight-forward in its explanation as others (such as wheat and barley).<br><br>So regarding the origin of your samples: Since when are the different accessions maintained in the collection? Do you have insights into the collection history? How is potential cross-pollination handled?<br><br>All the best and I am very much looking forward to dive deeper into the history of oat cultivation<br><br>Response: Thank you very much for your comments.<br><br>The collection and organization of oat germplasm resources in China began in the 1950s, led by the national agricultural ministry and the Crop Science Institute of the Chinese Academy of Agricultural Sciences. Several major collection and solicitation efforts were carried out [1].<br><br>Firstly, starting from the 1950s, relevant departments were organized by the national

agricultural ministry to carry out collection and recording work nationwide. The Crop Science Institute of the Chinese Academy of Agricultural Sciences also employed dedicated personnel to collect, organize, and preserve oat germplasm resources domestically and internationally, while agricultural research units in North China successively carried out the collection and organization of local farmer varieties. By the late 1950s, the Crop Science Institute of the Chinese Academy of Agricultural Sciences imported 489 oat germplasm resources from 21 countries including the Soviet Union, Canada, Sweden, France, Denmark, Mongolia, Hungary, and Japan. By 1966, a total of 1,497 domestic and foreign oat germplasm resources were collected, organized, and registered [2].

Secondly, with the resumption of foreign introduction work in 1973, the Crop Science Institute of the Chinese Academy of Agricultural Sciences carried out the second oat germplasm resource introduction and collection work from the 1970s to the 1990s. According to the "Catalogue of Chinese Oat Germplasm Resources I" [3] compiled in the 1980s and the "Catalogue of Chinese Oat Germplasm Resources II" [4] compiled in the 1990s, a total of 2,978 oat resources were included, covering nine species of oat. At the beginning of the 1980s, three levels of seed banks were established at the national, regional, and major producing province (region) levels in China. At the same time, wild oat resources in North China, Northwest China, Southwest China, and Tibet were investigated and identified.

Thirdly, since the 1990s, with the increasing attention to oat research and strengthened national cooperation, introduction and collection work has been carried out gradually. Through cooperation with the Plant Gene Resources of Canada (PGRC), wild oat and local variety resources have been collected from around the world. Currently, China has collected and preserved over 6,200 oat germplasm resources, including 29 species [5].

Oats are a typical self-pollinating plant that generally undergoes self-fertilization in the absence of human intervention, but natural hybridization exists between different materials [6-8]. The structure of oat flowers determines its low natural outcrossing rate. We applied for the most primitive preserved seeds from the germplasm bank, which were very limited in quantity and needed to be multiplied. To prevent contamination and natural hybridization during propagation, the single seed descents (SSD) method was used, where one seed is grown, protected from out-crossing, and then one seed from the progeny is regrown, and this process is repeated for several generations until enough seeds are obtained for molecular experiments and phenotypic studies.

We completely agree with your viewpoint that demographic history influences the inference of genetic diversity, divergence time, and other conclusions. However, due to the limited data currently available, we are unable to draw more in-depth conclusions about the domestication history of oats. For example, to study gene flow, we need more outgroup genomic information; To infer population demographic history, we require larger-scale population resequencing data for accurate SNP phasing (We have performed MSMC analysis on several hulled and naked oat genomes with high sequencing depth, but the results, whether for all chromosomes or for each of the three subgenomes separately, did not yield any meaningful information.). We have already begun to de novo assemble genomes of several representative hexaploid related species of oats and representative hulled and naked oats, as well as conducting larger-scale whole-genome resequencing. We hope that with these data, we can gain a more thorough understanding of the domestication history of oats. Accordingly, we have made relevant explanations in the Discussion section (Line 341-352). Thank you for your valuable suggestions.

[1] Zheng, D., and Zhang, Z. (2017). Introduction and Utilization of Foreign Oat Germplasm Resources in China. Journal of Plant Genetic Resources 18, 1001–1005. doi: 10.13430/j.cnki.jpgr.2017.06.001.
[2] Zhao, X., Rong, Y., and Zhao, L. (2007). Collection and Evaluation of Oat Germplasm Resources in China. Pratacultural Science 24, 36–40.
[3] Crop Germplasm Resources Institute, Chinese Academy of Agricultural Sciences (1984). Catalogue of Chinese Oat Germplasm Resources I. China Agriculture Press, 1-97.
[4] Crop Germplasm Resources Institute, Chinese Academy of Agricultural Sciences

(1996). Catalogue of Chinese Oat Germplasm Resources I. China Agriculture Press, 1-79.
[5] Peng, Y. (2019). Oat Germplasm Resources and Breeding and Cultivation Management in Southwest China. Chinese Agriculture Press, 17-18.
[6] Shorter, R., Gibson, P., and Frey, K. J. (1978). Outscoring Rates in Oat Species Crosses (Avena sativa L. × A. sterilis L.)1. Crop Science 18:877-878, cropsci1978.0011183X001800050051x. doi: 10.2135/cropsci1978.0011183X001800050051x.
[7] Grindeland, R. L., and Frohberg, R. C. (1966). Outcrossing of Oat Plants (Avena sativa L.) Grown from Mutagen-Treated Seeds1. Crop Science 6, cropsci1966.0011183X000600040031x. doi: 10.2135/cropsci1966.0011183X000600040031x.
[8] Yang, F., and Wang, Z. (2021). Genetic Diversity Analysis and Outcrossing Rate Estimation of Oat Based on SSR Molecular Markers. Molecular Plant Breeding.

Reviewer #2: 1. There are more We word or We also words in the manuscript. As the typical original research article, passive voice is the main expression model, it will be more accurate and smoother after revised the We word in the whole manuscript, especially in the Abstract.

Response: Thank you very much for your kind suggestion. We have modified most of the active voice into passive voice.

2. Line84-85 and line 101-102, 325 is repetition.

Response: The repetitive sentences have been modified.

3. The Latin names should be added in the bracket after hulled oat and naked oat while they present firstly.

Response: Thank you very much for your professional advice. We have made the modifications as requested.

4. The Avena nuda is the homotypic synonym of A.sativa subsp.nuda or A.sativa var.nuda, and the reference genome OT3098 is also from a species of A.sativa, the Latin name should be present in the manuscript.

Response: We have made the modifications as requested.

5. As the allohexaploid oat, its domestication is complicated, especially for that the evolution and differentiation of subgenomes might inconsistent. So the divergence times in Fig3f should be re-estimated based on Sub-genome A, Sub-genome C, Sub-genome D, respectively. The Kya in Fig3f could be replaced by Mya.
Response: Thank you for your suggestion. We have separately calculated the divergence time of the three subgenomes. The divergence time of subgenome A is 47.3 (34.6~59.8) Kya, subgenome C is 47.0 (32.9~62.1) Kya, and subgenome D is 53.3 (38.8~67.5) Kya. We also included this result in our manuscript. We confirmed again that the time unit is indeed Kya.

6. The sequence depth of three germplasms (R21, R22, R86) selected were above 20X, estimation of the effective population size (Ne) history by using PSMC or MSMC2 as supplementary figure might provide another evidence of naked oat domestication independently.

Response: Thank you for your suggestion. We performed MSMC analysis on several hulled and naked oat genomes with high sequencing depth, but the results, whether for all chromosomes or for each of the three subgenomes separately, did not yield any meaningful information. We are unsure if this is due to MSMC not being effective in polyploid species such as oat or for other reasons, and have therefore decided not to include these results in the manuscript.

	7. The means of brown and blue lines in Fig4a and Fig4b should be list in the legend.<br><br>Response: Thanks for your comment. We have added the statement "Brown lines represent shared derived alleles, while blue lines represent shared more derived alleles than expected due to introgression." to the figure legend.<br><br>8. The text and figures in results of GWAS was inconsistent.<br><br>Response: Thank you for reminding us. We are ashamed of our carelessness. The necessary corrections have been made.<br><br>9. The results of significance analysis should be list as label, especially for the histogram in Fig2c, Fig3d, Fig5f-5h, Fig6d, Fig7d.<br><br>Response: Thank you for your comment. Significant labels have now been added to Fig2c and Fig3d. Fig5f-5h, Fig6d, and Fig7d already had significant labels as they involve comparing more than three groups, so they are marked with letter notation at the top of the figures.<br><br>10. The source of seeds should be definite in the plant materials of Materials and Methods. The 115 accessions in the manuscript were suggested to replace as 115 germplasms after the seed was provided by NGBC or theses germplasms had accessed as in NGBC, it might be misunderstood as these data were download from public database by the accessions were overmentioned.<br><br>Response: Thank you for your comment. We have added an explanation about the source of our seeds. However, we still use "accession" to represent different oat varieties because it not only denotes the registration number of the sequencing data but also identifies the germplasm resources in the germplasm bank. We believe that this will not cause any confusion, as in many other publications (e.g., [1-3]), accession is also used to represent different varieties, even for data generated by the studies themselves.<br><br>1. Cai, X., Sun, X., Xu, C., Sun, H., Wang, X., Ge, C., Zhang, Z., Wang, Q., Fei, Z., Jiao, C., et al. (2021). Genomic analyses provide insights into spinach domestication and the genetic basis of agronomic traits. Nat Commun 12, 7246. 10.1038/s41467-021-27432-z.<br>2. Yu, Y., Guan, J., Xu, Y., Ren, F., Zhang, Z., Yan, J., Fu, J., Guo, J., Shen, Z., Zhao, J., et al. (2021). Population-scale peach genome analyses unravel selection patterns and biochemical basis underlying fruit flavor. Nat Commun 12, 3604. 10.1038/s41467-021-23879-2.<br>3. Liu, Y., Du, H., Li, P., Shen, Y., Peng, H., Liu, S., Zhou, G.-A., Zhang, H., Liu, Z., Shi, M., et al. (2020). Pan-Genome of Wild and Cultivated Soybeans. Cell. 10.1016/j.cell.2020.05.023.<br><br>11. The heatmap of SNP density in Fig2A was suggested to present as single color with 3 gradients, such as blues-3-seq or greens-3-seq. The π= or Fst= was suggest to add before the numbers.<br><br>Response: Thank you for your comment. We have followed your suggestion and added "π=" and "Fst=" before the numbers. However, we insist on using the original colors in the heatmap because the color scheme from cool (blue) to warm (red) is the most commonly used color scheme for heatmaps.
**Additional Information:**	
**Question**	**Response**
Are you submitting this manuscript to a special series or article collection?	No

Powered by Editorial Manager® and ProduXion Manager® from Aries Systems Corporation

**Experimental design and statistics**<br><br>Full details of the experimental design and statistical methods used should be given in the Methods section, as detailed in our Minimum Standards Reporting Checklist. Information essential to interpreting the data presented should be made available in the figure legends.<br><br>Have you included all the information requested in your manuscript?	Yes
**Resources**<br><br>A description of all resources used, including antibodies, cell lines, animals and software tools, with enough information to allow them to be uniquely identified, should be included in the Methods section. Authors are strongly encouraged to cite Research Resource Identifiers (RRIDs) for antibodies, model organisms and tools, where possible.<br><br>Have you included the information requested as detailed in our Minimum Standards Reporting Checklist?	Yes
**Availability of data and materials**<br><br>All datasets and code on which the conclusions of the paper rely must be either included in your submission or deposited in publicly available repositories (where available and ethically appropriate), referencing such data using a unique identifier in the references and in the "Availability of Data and Materials" section of your manuscript.<br><br>Have you have met the above requirement as detailed in our Minimum Standards Reporting Checklist?	Yes

Powered by Editorial Manager® and ProduXion Manager® from Aries Systems Corporation

Manuscript

Click here to access/download;Manuscript;maintext.revised.docx

Genome resequencing reveals independent domestication and breeding improvement of naked oat

Jinsheng Nan[1#], Yu Ling[1#], Jianghong An[1,2#], Ting Wang[1], Mingna Chai[1], Jun Fu[24], Gaochao Wang[24], Cai Yang[3], Ting Wang[1], Mingna Chai[1], Yan Yang[1], Bing Han[1,2*]

[1] Key Laboratory of Germplasm Innovation and Utilization of Triticeae Crops at Universities of Inner Mongolia Autonomous Region, Inner Mongolia Agricultural University, Hohhot, China

[24] Beijing 8omics Gene Technology Co. Ltd, Beijing, China

[2] Inner Mongolia Academy of Agricultural & Animal Husbandry Sciences, Hohhot, China

[3] Inner Mongolia Guomai Agriculture Co., Ltd, Xilingol LeagueHohhot, China

[4] Beijing 8omics Gene Technology Co. Ltd, Beijing, China.

# These authors contributed equally: Jinsheng Nan, Yu Ling, Jianghong AnN

* Correspondence and requests for materials should be addressed to Bing Han (email: hb_nmg@163.com)

Email:

Jinsheng Nan: 470917658@qq.com
Yu Ling: lingyuolcuc@126.com
Jianghong An: 1910932116@qq.com
Jun Fu: coly_fu@163.com
Gaochao Wang: gaochaowang@ymail.com
Cai Yang: yangcai-china@163.com
Ting Wang: wangtinger0115@163.com
Mingna Chai: 1070703735@qq.com

Yan Yang: yangyanchutao@126.com
Bing Han: hb_nmg@163.com

Abstract

As an important cereal crop, common oat has attracted more and more attention due to its healthy nutritional components and bioactive compounds. Here, ~~we performed~~ high-depth resequencing of 115 oat accessions and closely related hexaploid species worldwide was performed. Based on genetic diversity and linkage disequilibrium analysis, it was~~we~~ found that hulled oat (*Avena sativa*) experienced a more severe bottleneck than naked oat (*Avena sativa var. nuda*). Combined with the divergence time of ~51,200 years ago, ~~we rejected~~ the previous speculation that naked oat was a variant of hulled oat was rejected. It was~~We~~ found that the common segments that hulled oat introgressed to naked oat cultivars contained 444 genes, mainly enriched in photosynthetic efficiency-related pathways. ~~We identified~~ S~~s~~elective sweeps during environmental adaptation and breeding improvement were identified in the naked oat genome. ~~We also identified c~~Candidate genes associated with smut resistance and the days to maturity phenotype were also identified. Our study provides genomic resources and new insights into naked oat domestication and breeding.

Formatted: Font: Italic

Formatted: Font: Italic

Keywords

Naked oat, Genetic diversity, Divergence time, Introgression, Selective sweep, GWAS

Introduction

Common oat (*Avena sativa*) ranks seventh in production among global cereal crops (http://www.fao.org/faostat/en/, accessed May 2022). It is one of the most important crops in several countries and is widely used as human food and animal feed [1]. Oats are high in protein,

Formatted: Font: Italic

rich in polyunsaturated fatty acids, and have a low carbon footprint [2]. In recent years, oat has attracted more and more attention as healthy food due to its rich content of various bioactive compounds, which can reduce the risk of cardiovascular diseases (CVD), type 2 diabetes mellitus (T2DM), gastrointestinal disorders, and cancer [3]. Oat is highly adaptable to a wide range of climates. Oat can be widely planted and have high yields in the harsh marginal environment where other major cereal crops, such as rice and corn, cannot be grown [4]. In China, naked oat (*Avena sativa var. nuda*) landraces are distributed from the warm and humid Yunnan-Guizhou region to the cold and dry Shanxi-Gansu region.

Common oat is typically classified into two types according to the morphology of the seeds: hulled and naked. Hulled oats are the widely known oats grown all over the world, while naked oat is grown mainly in China [5,6], and the most extensive germplasm collection for naked oat is maintained at the National Germplasm Bank of China (NGBC, http://www.cgris.net). Hulled oat has a caryopsis tightly surrounded by a thick, lignin-rich hull that remains attached to the mature grain throughout threshing and cleaning (Figure 1a). In contrast, naked oat is characterized by papery, free-threshing hulls that are mostly lost during threshing and cleaning [7]. Besides the free-threshing attribute, naked oat differs from hulled oat by having a multiflorous habit and elongated rachilla segments in the mature panicle [8].

Common oat is usually considered a secondary crop, i.e., derived from a weed of the primary cereal domesticates, wheat and barley [9]. Current thinking is that common oat was probably domesticated in central or northern Europe ~3000 years ago from a weedy hexaploid progenitor that may have been used as a forage crop [10]. There is as yet no consensus as to the origin of naked oat. It has been proposed that the naked oat is a separate species from the hulled oat, named *Avena nuda* L. [11]; it has also been proposed that the naked oat arose as a mutant of the domesticated hulled oat. The mutant theory suggested that naked oat may have originated from hulled oat, potentially after hulled oats reached China from its main center of diversity in

southwest Asia [2,12]. However, direct molecular evidence is scarce to be reported. It has been declared the genetic diversity of naked oat is less than that of hulled oat, which would support a bottleneck effect from a mutation event to domestication [7]. However, that study was based on genotyping by sequencing (GBS), and the number of markers used was relatively low (8,675 haplotype markers).

Genome sequencing can significantly accelerate functional genomic studies of crops [13]. Owing in part to the hexaploid nature of the common oat genome (AACCDD, 2n = 6x = 42), its very large size (~12 G), and its abundance of repetitive sequences, the genomes of hulled and naked oat have not been assembled until recently, long after other crop species such as rice, maize, and soybean. And there have been no large-scale resequencing studies of common oat to date. The recently published two high-quality genomes [2,14] of oat will indeed trigger the climax of oat functional genome research.

~~China is the main planting area and genetic diversity center of naked oat, and the NGBC has preserved 3,255 hulled and naked oat germplasm accessions.~~ Based on 455 previously defined accessions comparing the oat core collection of the NGBC [15], genetic diversity, and origin, we performed deep resequencing of 89 naked oat, 22 hulled oat, and four closely related hexaploid species from around the world. Our Bayesian analysis of divergence time support that hulled oat and naked oat diverged ~51,200 years ago, well before the domestication of oat. Moreover, the genetic diversity of naked oat is higher than that of hulled oat. Combined with other lines of evidence, we speculate that hulled oat and naked oat were domesticated independently. We also found that genetic introgression of hulled oat into naked oat cultivars increased their yield. We also investigated the genes selected during the environmental adaptation of naked oat landraces and the breeding of naked oat cultivars. Finally, we performed genome-wide association studies for two traits: oat smut resistance and days to maturity.

Results

Genetic diversity of cultivated oat

There are 3,255 oat accessions in the National Germplasm Bank of China (NGBC, http://www.cgris.net). From among the previously defined 455 core accessions [15], 189 accessions were selected by geographic origin and subjected to low-depth whole-genome resequencing using the BGI DNBSEQ-T7 platform (average of 46.13 G bases per sample, corresponding to 3.47 X coverage depth, Table S1). ~~We aligned t~~The sequencing data was aligned to the OT3098 reference genome (*Avena sativa*) [14] and ~~called~~ SNPs were called using BCFtools. Based on the detected SNPs, a phylogenetic tree (Figure 1b) and PCA (Figure 1c) were conducted, and 115 accessions were screened by maximizing the genetic diversity and geographic origin (Figure 1d, Table S2) for deep sequencing (average sequencing depth 13.17 X, Table S1), including 89 naked oats (81 landraces (“ONL”), eight cultivars (“ONC”)), 22 hulled oats (“OH”), and four closely related hexaploid species (*A.fatua* L., *A.occidentalis* Dur., *A.sterilis* L. and *A.byzantina* Koch.).

The average Q20 and Q30 ratios of sequenced data were 97.92% and 93.29% (Table S2), indicating the very high quality of the data. The average mapping rate was 99.30%, and the average coverage rate was 94.96% (Table S1). A total of 336,725,135 SNPs were detected in the 115 accessions. SNPs with low quality and MAF < 0.05 and missing rate > 20% were filtered out, leaving 52,817,822 SNPs for subsequent analyses (Figure 2a). The transitions vs. transversions ratio of the SNPs was 1.93, which was very close to the expected value of 2, indicating the high quality of the SNPs set.

Calculating the nucleic acid diversity (π) and genetic distance (Fst) of the ONL, ONC, and OH sets of accessions (Figures 2a and 2b) showed that the extent of polymorphism was highest in the ONL accessions (π=1.23e^{-3}), followed by ONC (π=1.12e^{-3}), and then OH (π=1.03e^{-3}). The genetic distance between ONL and OH is the farthest (Fst=0.116), and the genetic distance between ONC and ONL/OH is similar (Fst=0.085 vs. Fst=0.082). This may be related to the breeding history of hulled-naked hybridization in ONC [16].

~~We conducted a~~The runs of homozygosity (ROH) analysis was conducted, which indicated the following order for the degree of inbreeding (highest first): ONC, OH, ONL, and the closely related hexaploid species (OG) (Figure 2c). We detected strong linkage disequilibrium (LD) in the oat genome (Figure 2d) and noted that the r^2 of LD decay was greater than 0.4 for windows larger than 1Mb in all populations. This level of LD was much higher than in other crops such as rice [17], maize [18,19], and sorghum [20,21]. Because cross-breeding would lead to increased LD [22], the LD of ONC was much higher than that of OH and ONL.

Differentiation of hulled oat and naked oat

Although early studies proposed that naked oat was a separate species [11] (*A. nuda* L.), researchers now generally regard naked oat as a variant of hulled oat, potentially resulting from a mutation that occurred after hulled oat was introduced into China [2,7]. A phylogenetic tree clustered hulled oat and naked oat landraces as two independent clades (Figure 3a). We named these two separate clades OHc (n=19) and ONLc (n=57), respectively. Some accessions didn’t cluster according to the hulled-naked phenotype, reflecting cross-breeding. ADMIXTURE analysis (Figure 3b) found that the cross-validation error rate was the smallest when K=4 (Figure 3c). At this level, the accessions were clustered as ONLc, OHc, cross-breeding group, and OG. The cross-breeding group included all ONC and 15 ONL, indicating that naked oat landraces were also improved by crossing with hulled oat. Seven of the 9 ONL collected from outside China were clustered in the cross-breeding group.

~~W~~It was~~e~~ mentioned above that the genetic diversity of ONL was higher than that of OH. To avoid the effects of gene flow and sample size, ~~we performed~~ 100 random samplings (n=19, consistent with the sample size of OHc) of ONLc were performed to calculate genetic diversity. We found that the genetic diversity of ONLc was still higher than that of OHc ($1.08e^{-3}$ vs. $0.90e^{-3}$). Although the mapping rates of ONL and OH were high and not significantly different (99.04% vs. 99.92%, Figure 3d), the mapping rates of ONL had greater variation (standard deviation: 1.91 vs. 0.02). The PCA results showed that ONL occupied the largest variable space (Figure 3e), consistent with the genetic diversity finds. These contradicted the speculation that the naked oat is a variant of the hulled oat, in which the naked oat would experience an additional bottleneck, resulting in lower genetic diversity than the hulled oat. Moreover, the hulled oat had a stronger short-distance LD (Figure 2d), indicating that the hulled oat experienced a more severe bottleneck than the naked oat. Combining hulled oat and naked oat had a deep split on the phylogenetic tree, these lines of evidence argue against the idea that naked oats originated as a variant of hulled oats.

T~~We also modeled t~~he differentiation time of hulled oat and naked oat was also modeled. To calculate the divergence time between hulled oat and naked oat, ~~we extracted~~ all high-quality 4dtv loci genotypes in the whole genome were extracted. Through these neutral evolutionary sequences and using a calibration time (~0.78 MYA) based on the closely related hexaploid species *A. fatua* and cultivated oats on TIMETREE, the differentiation time of hulled oat and naked oat was predicted as ~51,200 years ago (Figure 3f). We have also separately calculated the divergence times of the three subgenomes. The divergence time of subgenome A was ~47.3 Kya, subgenome C was ~47.0 Kya, and subgenome D was ~53.3 Kya. This time predates the domestication of wheat ~10,000 years ago [23,24]. Cultivated oat was domesticated as a field weed of wheat [9], indicating that hulled oat and naked oat had diverged before domestication and underwent an independent domestication process.

Introgression from hulled oat into naked oat cultivars

The breeding of naked oat in China has gone through phases, including the direct collection and utilization of landraces, cross-breeding between naked oat varieties, and cross-breeding of naked oat with hulled oat [16]. In particular, the cross-breeding between hulled oat and naked oat is recognized to have increased the yield of naked oat by more than 30 % [16]. The national average yield increased from 75 kg/mu in 1998 to 150 kg/mu in 2014 [25]. Consistently, the ADMIXTURE and phylogenetic tree analyses supported the cross-breeding history of ONC. In addition, A Patterson's D statistical analysis (Figure 4a) indicated that ONC and OHc accessions share more derived alleles than would be expected by chance (D=-0.0207, Z-score=-15.23). The Patterson's D statistical analyses even indicated that ONC and OHc shared more derived alleles than ONLc (D=-0.0033 vs. D=-0.0207, Figure 4a and 4b).

To investigate the common introgression segments that OHc has contributed to ONC, thewe performed an rIBD (relative identical by descent) analysis was performed [26,27]. This predicted the total length of the OHc segments introgressed into ONC as 309 Mb (2.8 % of the whole genome) (Figure 4c). The majority (76.6%) of these introgressed segments are on chromosomes 2C, 3C, 4A, 4C, 4D, 5C, and 7C and contain a total of 444 predicted ORFs (Table S3). Note that the total length of introgressed segments in subgenome C was 4.63 and 4.24 times that of subgenome A and D, respectively (Figure 4d). Exploratory analyses using GO and KEGG indicated that this set of introgressed genes is enriched for annotations related to photosynthesis (Figure 4e and 4f, Table S4 and S5), suggesting that hulled oat may have contributed to yield improvements for naked oat cultivars by altering photosynthetic efficiency.

Among these 444 introgressed genes, many predicted gene products could be possibly related to differential yield, including, for example, a VIN3-like protein (Pepsico2_Contig10032), ABC transporters (Pepsico2_Contig7110, Pepsico1_Contig27635, Pepsico2_Contig16075, Pepsico1_Contig16853), a polypyrimidine tract-binding protein homolog 1-like

(Pepsico2_Contig20359), a BTB/POZ domain-containing protein (Pepsico2_Contig17276), cytochrome P450 enzymes (Table S6), a GDSL esterase/lipase (Pepsico2_Contig5911), bZIP transcription factors (Table S6), and light-harvesting chlorophyll a/b binding proteins (Table S6).

Environmental adaptability of naked oat landraces and artificial selection of cultivars

Oat is highly adaptable to various climates, including arid and cold regions, and is an excellent species for studying crop abiotic stress tolerance [28,29]. Studying the environmental adaptability of oat can point toward genetic mechanisms and can also provide guidance for the genetic improvement of oat. To study the environmental adaptation mechanisms of naked oats, we used two clades of ONL on the phylogenetic tree: one for accessions from arid and low-temperature regions of China, including Gansu, Ningxia, and Qinghai (Group GNQ), and one for accessions from the Yunnan, Sichuan, and Guizhou (Group YG), three provinces with relatively higher rainfall and temperature (Tables S7).

The annual rainfall, average temperature, frost-free period, and accumulated temperature of YG were significantly higher than that of GNQ by Student’s t-test: the annual rainfall (1203.75 mm vs. 440.00 mm, *P*=0.016, Figure 5a), the annual average temperature (16.67 °C vs. 8.02 °C, *P*=0.00049, Figure 5b), frost-free period (236.5 days vs. 151.82 days, Figure 5c), and accumulated temperature (5556.0 °C vs. 2684.4 °C, *P*=0.0025, Figure 5d). ~~We performed~~ A~~a~~ selective sweep analysis was performed that predicted 8,620 selective sweep signatures in the group GNQ when using the group YG as a reference (Figure 5e). Notably, a gene enrichment analysis indicated that the genes within the selective sweep regions were enriched for functional annotations related to drought resistance, cold acclimation, and DNA damage repair (Table S8 and S9).

Naked oat breeding programs have successfully improved cultivars' yield and lodging resistance [16,25]. We investigated yield-related agronomic traits, including spikelet number, grain number per spike, spikelet length, panicle length, and grain weight per spike for OH, ONL, and ONC (Table S10). ~~We~~ It was found that ONC was significantly improved over ONL among all these phenotypes ($P < 0.01$, Student's t-test). In particular, spikelet number (Figure 5f), grain number per spike (Figure 5g), and grain weight per spike (Figure 5h) were greatly improved.

Seeking to identify genes that were selected during the improvement of ONC, we performed a selective sweep analysis using ONLc as a reference (Figure 5g). ~~We predicted~~ A~~a~~ total of 7,667 selective sweep signatures were predicted. The set of genes within the putatively selected regions was enriched for annotations, including "carbohydrate mediated signaling", "sugar mediated signaling pathway", and "regulation of developmental vegetative growth" (Table S11 and S12). Some of the selected genes may be related to cultivars' improved yield and lodging resistance. For example, previous studies have reported that cellulose synthase enzymes can improve the lodging resistance of oat stems by increasing the content of the stem structural carbohydrate cellulose [30,31]. There were 30 genes related to cellulose synthesis among the genes from the candidate selective sweep regions (Table S13). Many yield-related genes were among the candidate regions, such as *AP2* (13 genes were annotated as *AP2*, Table S12). *APETALA 2* (*AP2*)-like family plays an essential role in inflorescence and spikelet development [32]. These candidate genes can provide helpful information for future oat improvement.

Association analysis between smut resistance of oat and days to maturity

Oat smut is a major oat disease caused by fungal pathogens of the family Ustilaginaceae [33]; it affects the heading stage and seriously reduces oat yields [34]. To enable an association analysis, ~~we downloaded~~ phenotypic data of the 115 accessions (Table S2) was downloaded

from the Chinese Crop Germplasm Resources Information System (CCGRIS, http://www.cgris.net). The phenotype data was collected in experiments that used uniform strains and inoculation methods, and three-year averages were taken. Using the detected 52,817,822 SNPs, we performed a genome-wide association study (GWAS) and identified a significant association signal on oat chromosome 2D (Figure~~.~~ 6~~1~~a~~A~~). Five predicted ORFs contained significant SNPs, or were located upstream and downstream of significant SNPs. A gene (Pepsico2_Contig5200) positioned 43 Kb from the most significant SNP was annotated as Zealexin A1 synthase (Figure 6~~1~~b). In maize, CYP71Z18 catalyzes the formation of maize phytoalexins, including zealexin A1. Overexpression of CYP71Z18 in rice resulted in the accumulation of several new diterpenoids, and transgenic rice also showed more strong resistance to rice blast infection [35]. There are 31 SNPs within the gene and a 2 Kb upstream region, of which 3 SNPs are synonymous mutations: one is located in an intron, and one is located in the 3'-UTR. The 31 SNPs constituted two haplotypes, with Hap.1 containing 91 accessions and Hap.2 containing 12 accessions (Figure 6~~1~~c). All accessions carrying Hap.1 were susceptible to smut infection, while all accessions carrying Hap.2 had some degree of smut resistance (Figure 6~~1~~d). Hap. 1 can be used as a reliable molecular marker for marker-assisted selection (MAS) to develop new smut-resistant varieties.

An agronomic trait known as days to maturity (DTM) describes the average number of days from planting until harvest. For a given species, in general, the longer the DTM, the higher the yield [36]. To obtain candidate genes associated with DTM, ~~we performed~~ GWAS was performed using the SNPs mentioned above and phenotypic data for the 115 accessions downloaded from the CCGRIS. These phenotypic data were the mean of 3-year measurements in origin or adapted ecoregion, i.e., under normal growth conditions. Association analysis found a significant association signal on oat chromosome 4C (Figure~~.~~ 7~~1~~a~~A~~). 11 predicted ORFs contained significant SNPs, or were located upstream and downstream of significant

SNPs. Among them, Pepsico1_Contig35784.mrna1 has been annotated as a pentatricopeptide repeat-containing protein (PPR) (Figure 7b). A previous study showed that loss-of-function of this gene promoted early flowering in Arabidopsis [37]. There are 3 SNPs in the gene: one located 2 kb upstream of the gene, one located in the 5'-UTR region, and another in an exon that is predicted to cause a missense mutation. These three SNPs constitute three haplotypes (Figure 7c). Ninety-six accessions were carrying Hap. 1 with an average DTM of 103.6 days; 9 accessions were carrying Hap.2 with an average DTM of 182.7 days; and five accessions were carrying Hap.3 with an average DTM of 118.2 days (Figure 7d).

Discussion

A total of ~~We collected~~ 115 oat accessions worldwide, including 89 naked oats (81 landraces and eight cultivars), 22 hulled oats, and four closely related hexaploid species were collected. ~~We calculated~~ T~~t~~he genetic diversity of each oat population was calculated based on high-depth sequencing data for these 115 accessions. It was~~We~~ found that the genetic diversity of naked oat was higher than that of hulled oat (1.23e^{-3} vs. 1.12e^{-3}). This is contrary to previous reports [2,7] and could reflect the low number of markers used in those studies (8,675 haplotype markers). We found considerable differences in genetic diversity in different regions of chromosomes, such as ONLc, the mean π of chromosomes with the largest and smallest genetic diversity were 0.47e^{-3} and 2.45e^{-3} (Figure 2a), respectively. Therefore, a small number of markers may lead to sampling bias.

Moreover, compared with naked oat, hulled oat had stronger short-distance linkage disequilibrium. These pieces of evidence suggested that hulled oat experienced a more severe bottleneck than naked oat, which contradicts the previously reported speculation that naked oat originated as a variant of hulled oat [7]. If naked oat is a variant of hulled oat, naked oat would experience an additional bottleneck after the domestication bottleneck shared with hulled oat. By calculating the divergence time of hulled oat and naked oat, we estimate that these two oat types differentiated ~51,200 years ago, much earlier than the estimated domestication time of ~3000 years ago for oat [10]. Distinct from multiple previous proposals [2,7], these lines of evidence from our study suggest that hulled oat and naked oat were domesticated independently.

Crop domestication and improvement history, however, is a complex research topic. Like other crops such as rice [38–42]~~[1]~~, maize [43–47], and wheat [48–51], it often requires more genomic data as well as archeological and geographical evidence. Therefore, the conclusions drawn from our article based on existing data may have certain limitations. For instance, the lack of more genomic data from closely related hexaploid oats limits our understanding of gene flow in domestication and improvement [52]~~[1–5]~~. More research is needed to further reveal the clear outline of oat domestication and improvement history.

Through the efforts of breeders in recent decades, the yield of naked oat has been dramatically improved [16,25]. The way to achieve this includes a continuous artificial selection of landraces and cross-breeding with hulled oat. We found genetic evidence for both ways. Through selective sweep analysis of ONC, we identified a large number of selective signatures of genes related to yield and lodging resistance. The cross-breeding history has also been revealed by the Patterson's D statistic and rIBD analyses. The common introgressed segments from hulled oat in OHC contained 444 genes enriched for annotations related to photosynthesis. Compared with ONL, yield-related traits of ONC mainly improved spikelet number, grain

number per spike, and grain weight per spike. In addition, our GWAS results provided MAS markers for future genetic improvement of oat.

There is a large amount of arid and semi-arid land worldwide [53]. Research on the genetic mechanism of drought resistance of crops can help to develop new varieties of drought-resistant crops and promote human food security [54,55]. Oat is often grown in harsh, semi-arid environments, making them an excellent species to study the genetic mechanisms of drought resistance [4]. Candidate genes associated with drought resistance were obtained through selective sweep analysis using naked oat landraces from arid environments. For example, among the candidate genes, there were 43 UDP-glucosyltransferases. A previous study reported that overexpression of UDP-glycosyltransferase 3 (*UGT3*) can enhance rice's drought and salt stress tolerance [56]. In Arabidopsis, over-expression of *UGT79B2/B3* significantly enhanced plant tolerance to low temperature as well as drought and salt stress, whereas *ugt79b2/b3* double mutants generated by RNAi and CRISPR-Cas9 were more susceptible to adverse environmental conditions [57]. These candidate genes can provide helpful information for future studies on oat drought resistance.

In summary, our study provides valuable genomic resources for oat genomic and genetic research. We have overturned proposals about the origin of the naked oat and raised the idea that the naked oat was independently domesticated. Through introgression, selective sweep, and GWAS analyses, we provide a genomic framework and valuable information for facilitating marker-assisted selection for oat breeding.

Materials and Methods

Plant materials

There are 3,255 oat accessions in the National Germplasm Bank of China. From among the previously defined 455 accessions comprising the core collection [15], 189 accessions were selected based on their geographic origin. TheWe sowed seeds of the 189 selected accessions mentioned above were sowed in a seedling tray at a depth of 2 cm, keeping the soil moist. When the oats grew to the 3-leaf stage, we collected leaves from three plants for each accession, mixed the samples, placed them in 2 mL centrifuge tubes, and immediately stored them in liquid nitrogen. The sample source map was visualized using the Python package folium. The background map used is from Stamen Design (http://stamen.com).

Whole genome resequencing

Genomic DNA was extracted from young leaves using DNeasy Plant Mini Kits (Qiagen GmbH, Hilden, Germany). The quality and concentration of DNA were assessed by 1.0% agarose gel electrophoresis and using a nanodrop spectrophotometer (Thermo Fisher Scientific, Waltham, MA, USA). Sequencing library construction was performed using the MGIEasy FS PCR-Free DNA Library Prep Set (MGI Tech, Shenzhen, China), following the manufacturer's instructions. DNA sequencing was performed using the 2 x150 bp paired-end mode of the DNBSEQ-T7 platform (MGI Tech, Shenzhen, China, RRID:SCR_017981).

SNP calling

The quality of the generated sequencing data was assessed using fastp (RRID:SCR_016962) [58], and high-quality reads were aligned to the OT3098 reference genome (https://wheat.pw.usda.gov/GG3/graingenes_downloads/oat-ot3098-pepsico) using BWA mem (RRID:SCR_010910) [59] with the default parameters. SAMtools fixmate (RRID:SCR_002105) [60] was used to fill in the mate coordinates, insert sizes, and mate

related flags. PCR duplicates were marked using SAMtools markdup. BCFtools (RRID:SCR_005227) [61] was used to perform joint SNP calling. To reduce false positives, genotypes with quality less than 10 were marked as missing, and SNPs with 'QUAL < 30 | INFO/FS > 60.0 | INFO/MQ < 40.0 | F_PASS(GQ>=10 & GT!="mis") < 0.8 | MAF < 0.05' were filtered out. Gene-based SNP annotation was performed using the SnpEff (RRID:SCR_005191) [62] software package.

Phylogenetic tree and population structure analyses

An identity by state (IBS) distance-based neighbor-joining tree was built using the bionj function of the R package ape (RRID:SCR_017343) [63]. The pairwise IBS distances of all oat accessions were estimated using PLINK (RRID:SCR_001757) [64]. A model-based ancestry estimation was conducted using the program ADMIXTURE (RRID:SCR_001263) [65] with K = 2 to 8. The cross-validation procedure was used to determine the K number of the best fit model. Principal component analysis (PCA) was performed using the smartPCA program implemented in the EIGENSOFT (RRID:SCR_004965) [66] package.

Genetic diversity and genetic distance analyses

Nucleotide diversity ($\theta\pi$, the average number of pairwise nucleotide differences per site between any two randomly chosen DNA sequences from the population) and fixation index (Fst) across the whole genome were calculated using VCFtools (RRID:SCR_001235) [67] with a sliding window of 1 Mb and a step size of 500 Kb. Runs of homozygosity (ROH) segments were detected using PLINK (RRID:SCR_001757) [64] with the parameters "-homozyg-window-snp 50 --homozyg-window-missing 2 --homozyg-window-het 0 --homozyg-snp 50 --homozyg-kb 500 --homozyg-density 50".

Linkage disequilibrium decay

To estimate and compare linkage disequilibrium (LD) decay patterns, we used PopLDdecay (RRID:SCR_022509) [68] to calculate the mean squared correlation coefficient (r^2) values of all SNP pairs within 1 Mb. A bin size of 500 bp was used to generate the LD decay plot.

Divergence time estimation

To estimate the divergence time between hulled oat and naked oat, we identified all 6,814,874 four-fold degenerate loci (4dtv) according to the gene models of the oat reference genome gene annotations. These loci from three high-depth sequencing accessions (R86 from naked oat, R148 from hulled oat, and *A. fatua*) were then genotyped using BCFtools (RRID:SCR_005227) [61]. To obtain high-confidence genotypes, loci with QUAL less than 60 or GQ less than 30 in any accession were filtered out. Finally, 38,028 loci were left to estimate the divergence time. The divergence time was estimated using an uncorrelated relaxed clock in BEAST (RRID:SCR_010228) [69]. The divergence time between *A. fatua* and *A. sativa* acquired from TIMETREE (http://www.timetree.org, RRID:SCR_021162) was used to calibrate the evolutionary rate (Blosum62 and an uncorrelated exponential relaxed model). The Yule speciation process was used, which specifies a constant rate of species divergence. Normal priors were used for hulled oat and naked oat divergence time. The chain length was set to 10,000,000, sampling every 1,000 steps. Tracer (RRID:SCR_019121) [70] was used for visualizing and analyzing the Bayesian MCMC runs.

Introgression analysis

To detect introgression between naked oat cultivars and hulled oat, we used Patterson's D-statistic [71], which was implemented in the R package admixtools (RRID:SCR_018495) [72], to test gene flow with four hexaploid relatives as the outgroup. The significance was assessed by a block jackknife procedure. To clarify the genomic location of the candidate introgression

segments, we performed rIBD [73] analysis. First, we phased the genotypes using Beagle (RRID:SCR_001789) [74] and then used RefinedIBD [75] to detect shared IBDs tracks between any two accessions. We counted the number of shared IBD tracks with a 100 Kb sliding window and a 50 Kb step in comparing each naked oat cultivar with ONL or OH accessions. These counts were then normalized as nIBD = shared IBD number / number of ONL or OH), and the rIBD was calculated as rIBD = $nIBD_{OH} – nIBD_{ONL}$. rIBD values of all windows were calculated and then normalized following a standard normal distribution. Windows with Z-scores greater than 2 were considered as putative introgression segments.

Gene enrichment analysis

GO, and KEGG enrichment analyses of selected genes were performed using the R package ClusterProfiler (RRID:SCR_016884) [76]. The terms with an adjusted *P* value less than 0.05 were considered as significantly enriched.

Selective sweep analysis

Selective sweep analysis was performed using a reimplementation of the Python version (https://github.com/hardingnj/xpclr) of XPCLR [77], with a window size of 100 Kb and a step size of 20 Kb. Other parameters were set as: --rrate 1e-8 --ld 0.95 --phased --maxsnps 200 --minsnps 10. The windows with the top 5% XPCLR scores were considered as putative selective sweep regions.

Whole genome association study

A linear mixed model implemented in the GEMMA [78] software toolkit was used for the whole genome association study (GWAS). Bonferroni correction was used to control the false discovery rate (FDR) for multiple testing, with a significant level of 0.05 (α = 0.05). Linkage disequilibrium blocks were detected and visualized using PopLDdecay [68].

Data Availability

The raw sequence data reported in this paper have been deposited into the China National GeneBank DataBase (CNGBdb) under accession number CNP0003840 (https://db.cngb.org/search/project/CNP0003840/), and the Sequence Read Archive of the National Center for Biotechnology Information (NCBI) under accession number PRJNA921897 (https://www.ncbi.nlm.nih.gov/bioproject/PRJNA921897).

Genome Sequence Archive [64] in National Genomics Data Center [65], China National Center for Bioinformation / Beijing Institute of Genomics, Chinese Academy of Sciences (GSA: PRJCA012239) that are publicly accessible at https://ngdc.cncb.ac.cn/gsa.

Abbreviations

GWAS: Whole Genome Association Study; NGBC: National Germplasm Bank of China; GBS: Genotyping by Sequencing; ROH: Runs of Homozygosity; LD: Linkage Disequilibrium; PCA: Principal Component Analysis; MYA: Million Years Ago; ORF: Open Reading Frame; GO: Gene Ontology; KEGG: Kyoto Encyclopedia of Genes and Genomes; CCGRIS: Chinese Crop Germplasm Resources Information System; SNP: Single Nucleotide Polymorphism; UTR: Untranslated Region; MAS: Marker Assisted Selection; DTM: Days to Maturity;

Funding

We appreciate the funding support from National Key Research and Development Project of China (2022YFE0119800), Funding for Key Laboratory of Inner Mongolia Autonomous Region, Agriculture and Animal Husbandry Innovation Fund Project of Inner Mongolia Autonomous Region (2022CXJJN04), Major Science and Technology Project of Inner

Mongolia Autonomous Region (2021SZD0017), and Funds for Educational Development and Reform Platform of Inner Mongolia Autonomous Region (2100001184).

Acknowledgments

We were grateful to the Institute of Crop Sciences, Chinese Academy of Agricultural Sciences, for assistance in sample collection.

Competing Interests

The authors declare no competing interests.

Authors' Contributions

BH designed the research; JN, YL, JA, JF, and GW performed the data analysis; JN, JA, and TW conducted the field experiments; BH, JN, YL, and JA wrote the paper. All authors read and approved the final manuscript.

References

1. Rasane P, Jha A, Sabikhi L, Kumar A, Unnikrishnan VS. Nutritional advantages of oats and opportunities for its processing as value added foods - a review. *J Food Sci Technol*. 2015; doi: 10.1007/s13197-013-1072-1.

2. Peng Y, Yan H, Guo L, Deng C, Wang C, Wang Y, et al.. Reference genome assemblies reveal the origin and evolution of allohexaploid oat. *Nat Genet*. 2022; doi: 10.1038/s41588-022-01127-7.

3. Martínez-Villaluenga C, Peñas E. Health benefits of oat: current evidence and molecular mechanisms. *Current Opinion in Food Science*. 2017; doi: 10.1016/j.cofs.2017.01.004.

4. Wang T, Du Y-L, He J, Turner NC, Wang B-R, Zhang C, et al.. Recently-released genotypes of naked oat (Avena nuda L.) out-yield early releases under water-limited conditions by greater reproductive allocation and desiccation tolerance. *Field Crops Research*. 2017; doi: 10.1016/j.fcr.2017.01.017.

5. Liu L, Ma M, Liu Z, Zhang L, Zhou J. Community structure of fungal pathogens causing spikelet rot disease of naked oat from different ecological regions of China. *Sci Rep*. Nature Publishing Group; 2021; doi: 10.1038/s41598-020-80273-6.

6. Nations F and AO of the U. Fodder Oats: A World Overview. Food & Agriculture Org.;

7. Yan H, Zhou P, Peng Y, Bekele WA, Ren C, Tinker NA, et al.. Genetic diversity and genome-wide association analysis in Chinese hulless oat germplasm. *Theor Appl Genet*. 2020; doi: 10.1007/s00122-020-03674-1.

8. Ougham HJ, Latipova G, Valentine J. Morphological and biochemical characterization of spikelet development in naked oats (Avena sativa). *New Phytologist*. 1996; doi: 10.1111/j.1469-8137.1996.tb01141.x.

9. Zhou X, Jellen EN, Murphy JP. Progenitor Germplasm of Domisticated Hexaploid Oat. *Crop Science*. 1999; doi: 10.2135/cropsci1999.0011183X003900040042x.

10. Vavilov NI, Vavylov MI, Dorofeev VF. Origin and Geography of Cultivated Plants. Cambridge University Press;

11. Zheng D, Zhang Z. Discussion on the Origin and Taxonomy of Naked Oat (Avena nuda L.). *Journal of Plant Genetic Resources*. 12:667–702011;

12. Loskutov IG. On evolutionary pathways of Avena species. *Genet Resour Crop Evol*. 2008; doi: 10.1007/s10722-007-9229-2.

13. Jia M, Guan J, Zhai Z, Geng S, Zhang X, Mao L, et al.. Wheat functional genomics in the era of next generation sequencing: An update. *The Crop Journal*. 2018; doi: 10.1016/j.cj.2017.09.003.

14. Kamal N, Tsardakas Renhuldt N, Bentzer J, Gundlach H, Haberer G, Juhász A, et al.. The mosaic oat genome gives insights into a uniquely healthy cereal crop. *Nature*. 2022; doi: 10.1038/s41586-022-04732-y.

15. Zhang E, Zhang Z, Wang T, Li Y, Wu B. Studies on Sampling Strategies to Develop Core Collection of Chinese Oat Ger mplas m. *Journal of Plant Genetic Resources*. 9:151–62008;

16. Yang X, Zhou H, Yang C, Zhang X, Li T. Review and development direction of oat breeding technology of China. *China Seed Industry*. :6–7 2012;

17. Huang X, Wei X, Sang T, Zhao Q, Feng Q, Zhao Y, et al.. Genome-wide association studies of 14 agronomic traits in rice landraces. *Nat Genet*. 2010; doi: 10.1038/ng.695.

18. Li C, Huang Y, Huang R, Wu Y, Wang W. The genetic architecture of amylose biosynthesis in maize kernel. *Plant Biotechnol J*. 2018; doi: 10.1111/pbi.12821.

19. Riedelsheimer C, Czedik-Eysenberg A, Grieder C, Lisec J, Technow F, Sulpice R, et al.. Genomic and metabolic prediction of complex heterotic traits in hybrid maize. *Nature Genetics*. 2012; doi: 10.1038/ng.1033.

20. Bekele WA, Wieckhorst S, Friedt W, Snowdon RJ. High-throughput genomics in sorghum: from whole-genome resequencing to a SNP screening array. *Plant Biotechnology Journal*. 2013; doi: 10.1111/pbi.12106.

21. Mace ES, Tai S, Gilding EK, Li Y, Prentis PJ, Bian L, et al.. Whole-genome sequencing reveals untapped genetic potential in Africa’s indigenous cereal crop sorghum. *Nat Commun*. Nature Publishing Group; 2013; doi: 10.1038/ncomms3320.

22. Slatkin M. Linkage disequilibrium — understanding the evolutionary past and mapping the medical future. *Nat Rev Genet*. 2008; doi: 10.1038/nrg2361.

23. Eckardt NA. Evolution of Domesticated Bread Wheat. *Plant Cell*. 2010; doi: 10.1105/tpc.110.220410.

24. Haas M, Schreiber M, Mascher M. Domestication and crop evolution of wheat and barley: Genes, genomics, and future directions. *Journal of Integrative Plant Biology*. 2019; doi: 10.1111/jipb.12737.

25. Ren C, Cui L, Yang C, Tian C, Fu X, Liu Y, et al.. Establishment and Application of High Efficient Breeding Technology System of Oat in China. *Journal of Agricultural Science and Technology*. 18:1–62016;

26. Wang X, Chen L, Ma J. Genomic introgression through interspecific hybridization counteracts genetic bottleneck during soybean domestication. *Genome Biology*. 2019; doi: 10.1186/s13059-019-1631-5.

27. Bosse M, Megens H-J, Frantz LAF, Madsen O, Larson G, Paudel Y, et al.. Genomic analysis reveals selection for Asian genes in European pigs following human-mediated introgression. *Nat Commun*. Nature Publishing Group; 2014; doi: 10.1038/ncomms5392.

28. Han L, Eneji AE, Steinberger Y, Wang W, Yu S, Liu H, et al.. Comparative Biomass Production of Six Oat Varieties in a Saline Soil Ecology. *Communications in Soil Science and Plant Analysis*. Taylor & Francis; 2014; doi: 10.1080/00103624.2014.912299.

29. Islam M, Eneji A, Z R, G H, G C, Xue X. Oat-based cropping system for sustainable agricultural development in arid regions of northern China. *Journal of Agriculture, Biotechnology and Ecology*. 3:1–82010;

30. Liu W, Deng Y, Hussain S, Zou J, Yuan J, Luo L, et al.. Relationship between cellulose accumulation and lodging resistance in the stem of relay intercropped soybean [Glycine max (L.) Merr.]. *Field Crops Research*. 2016; doi: 10.1016/j.fcr.2016.07.008.

31. Zhang R, Jia Z, Ma X, Ma H, Zhao Y. Characterising the morphological characters and carbohydrate metabolism of oat culms and their association with lodging resistance. *Plant Biol (Stuttg)*. 2020; doi: 10.1111/plb.13058.

32. Debernardi JM, Greenwood JR, Jean Finnegan E, Jernstedt J, Dubcovsky J. APETALA 2-like genes AP2L2 and Q specify lemma identity and axillary floral meristem development in wheat. *The Plant Journal*. 2020; doi: 10.1111/tpj.14528.

33. Feldbrügge M, Kellner R, Schipper K. The biotechnological use and potential of plant pathogenic smut fungi. *Appl Microbiol Biotechnol*. 2013; doi: 10.1007/s00253-013-4777-1.

34. Hu J, Yang JY, Li J, Gao T, Yang GW, Ren HY. First report of Ustilago avenae causing loose smut of oat (Avena sativa) in Shandong China. *J Plant Pathol*. 2018; doi: 10.1007/s42161-018-0016-z.

35. Shen Q, Pu Q, Liang J, Mao H, Liu J, Wang Q. CYP71Z18 overexpression confers elevated blast resistance in transgenic rice. *Plant Mol Biol*. 2019; doi: 10.1007/s11103-019-00881-3.

36. Li R, Li M, Ashraf U, Liu S, Zhang J. Exploring the Relationships Between Yield and Yield-Related Traits for Rice Varieties Released in China From 1978 to 2017. *Frontiers in Plant Science*. 102019;

37. Emami H, Kumar A, Kempken F. Transcriptomic analysis of poco1, a mitochondrial pentatricopeptide repeat protein mutant in Arabidopsis thaliana. *BMC Plant Biology*. 2020; doi: 10.1186/s12870-020-02418-z.

38. Chen E, Huang X, Tian Z, Wing RA, Han B. The Genomics of Oryza Species Provides Insights into Rice Domestication and Heterosis. *Annual Review of Plant Biology*. 2019; doi: 10.1146/annurev-arplant-050718-100320.

39. Civáň P, Craig H, Cox CJ, Brown TA. Three geographically separate domestications of Asian rice. *Nature Plants*. Nature Publishing Group; 2015; doi: 10.1038/nplants.2015.164.

40. Huang X, Han B. Rice domestication occurred through single origin and multiple introgressions. *Nature Plants*. Nature Publishing Group; 2015; doi: 10.1038/nplants.2015.207.

41. Huang X, Kurata N, Wei X, Wang Z-X, Wang A, Zhao Q, et al.. A map of rice genome variation reveals the origin of cultivated rice. *Nature*. Nature Publishing Group; 2012; doi: 10.1038/nature11532.

42. Li C, Zhou A, Sang T. Genetic analysis of rice domestication syndrome with the wild annual species, Oryza nivara. *New Phytologist*. 2006; doi: 10.1111/j.1469-8137.2005.01647.x.

43. Hufford MB, Xu X, van Heerwaarden J, Pyhäjärvi T, Chia J-M, Cartwright RA, et al.. Comparative population genomics of maize domestication and improvement. *Nat Genet*. Nature Publishing Group; 2012; doi: 10.1038/ng.2309.

44. Ramos-Madrigal J, Smith BD, Moreno-Mayar JV, Gopalakrishnan S, Ross-Ibarra J, Gilbert MTP, et al.. Genome Sequence of a 5,310-Year-Old Maize Cob Provides Insights into the Early Stages of Maize Domestication. *Current Biology*. 2016; doi: 10.1016/j.cub.2016.09.036.

45. Xu G, Lyu J, Li Q, Liu H, Wang D, Zhang M, et al.. Evolutionary and functional genomics of DNA methylation in maize domestication and improvement. *Nat Commun*. Nature Publishing Group; 2020; doi: 10.1038/s41467-020-19333-4.

46. Liu J, Fernie AR, Yan J. The Past, Present, and Future of Maize Improvement: Domestication, Genomics, and Functional Genomic Routes toward Crop Enhancement. *Plant Communications*. 2020; doi: 10.1016/j.xplc.2019.100010.

47. Hufford MB, Seetharam AS, Woodhouse MR, Chougule KM, Ou S, Liu J, et al.. De novo assembly, annotation, and comparative analysis of 26 diverse maize genomes. *Science*. American Association for the Advancement of Science; 2021; doi: 10.1126/science.abg5289.

48. THE INTERNATIONAL WHEAT GENOME SEQUENCING CONSORTIUM (IWGSC), Appels R, Eversole K, Stein N, Feuillet C, Keller B, et al.. Shifting the limits in wheat research and breeding using a fully annotated reference genome. *Science*. American Association for the Advancement of Science; 2018; doi: 10.1126/science.aar7191.

49. Maccaferri M, Harris NS, Twardziok SO, Pasam RK, Gundlach H, Spannagl M, et al.. Durum wheat genome highlights past domestication signatures and future improvement targets. *Nat Genet*. Nature Publishing Group; 2019; doi: 10.1038/s41588-019-0381-3.

50. Gaurav K, Arora S, Silva P, Sánchez-Martín J, Horsnell R, Gao L, et al.. Population genomic analysis of Aegilops tauschii identifies targets for bread wheat improvement. *Nat Biotechnol*. Nature Publishing Group; 2022; doi: 10.1038/s41587-021-01058-4.

51. Avni R, Nave M, Barad O, Baruch K, Twardziok SO, Gundlach H, et al.. Wild emmer genome architecture and diversity elucidate wheat evolution and domestication. *Science*. American Association for the Advancement of Science; 2017; doi: 10.1126/science.aan0032.

52. Mabry ME, Rowan TN, Pires JC, Decker JE. Feralization: Confronting the Complexity of Domestication and Evolution. *Trends in Genetics*. Elsevier; 2021; doi: 10.1016/j.tig.2021.01.005.

53. Huang J, Ji M, Xie Y, Wang S, He Y, Ran J. Global semi-arid climate change over last 60 years. *Clim Dyn*. 2016; doi: 10.1007/s00382-015-2636-8.

54. Hu H, Xiong L. Genetic Engineering and Breeding of Drought-Resistant Crops. *Annual review of plant biology*. 2013; doi: 10.1146/annurev-arplant-050213-040000.

55. Martignago D, Rico-Medina A, Blasco-Escámez D, Fontanet-Manzaneque JB, Caño-Delgado AI. Drought Resistance by Engineering Plant Tissue-Specific Responses. *Frontiers in Plant Science*. 102020;

56. Wang T, Ma Y, Huang X, Mu T, Li Y, Li X, et al.. Overexpression of OsUGT3 enhances drought and salt tolerance through modulating ABA synthesis and scavenging ROS in rice. *Environmental and Experimental Botany*. 2021; doi: 10.1016/j.envexpbot.2021.104653.

57. Li Y, Wang B, Dong R, Hou B. AtUGT76C2, an Arabidopsis cytokinin glycosyltransferase is involved in drought stress adaptation. *Plant Sci*. 2015; doi: 10.1016/j.plantsci.2015.04.002.

58. Chen S, Zhou Y, Chen Y, Gu J. fastp: an ultra-fast all-in-one FASTQ preprocessor. *Bioinformatics*. 2018; doi: 10.1093/bioinformatics/bty560.

59. Li H. Aligning sequence reads, clone sequences and assembly contigs with BWA-MEM. *arXiv*. 1303:3997v12013;

60. Li H, Handsaker B, Wysoker A, Fennell T, Ruan J, Homer N, et al.. The Sequence Alignment/Map format and SAMtools. *Bioinformatics*. 2009; doi: 10.1093/bioinformatics/btp352.

61. Li H. A statistical framework for SNP calling, mutation discovery, association mapping and population genetical parameter estimation from sequencing data. *Bioinformatics*. 2011; doi: 10.1093/bioinformatics/btr509.

62. Cingolani P, Platts A, Wang LL, Coon M, Nguyen T, Wang L, et al.. A program for annotating and predicting the effects of single nucleotide polymorphisms, SnpEff: SNPs in the genome of Drosophila melanogaster strain w1118; iso-2; iso-3. *Fly (Austin)*. 2012; doi: 10.4161/fly.19695.

63. Paradis E, Claude J, Strimmer K. APE: Analyses of Phylogenetics and Evolution in R language. *Bioinformatics*. 2004; doi: 10.1093/bioinformatics/btg412.

64. Purcell S, Neale B, Todd-Brown K, Thomas L, Ferreira MA, Bender D, et al.. PLINK: a tool set for whole-genome association and population-based linkage analyses. *American journal of human genetics*. 2007; doi: 10.1086/519795.

65. Alexander DH, Novembre J, Lange K. Fast model-based estimation of ancestry in unrelated individuals. *Genome research*. 2009; doi: 10.1101/gr.094052.109.

66. Patterson N, Price AL, Reich D. Population structure and eigenanalysis. *PLoS genetics*. 2006; doi: 10.1371/journal.pgen.0020190.

67. Danecek P, Auton A, Abecasis G, Albers CA, Banks E, DePristo MA, et al.. The variant call format and VCFtools. *Bioinformatics*. 2011; doi: 10.1093/bioinformatics/btr330.

68. Zhang C, Dong S-S, Xu J-Y, He W-M, Yang T-L. PopLDdecay: a fast and effective tool for linkage disequilibrium decay analysis based on variant call format files. *Bioinformatics*. Oxford Academic; 2019; doi: 10.1093/bioinformatics/bty875.

69. Bouckaert R, Heled J, Kühnert D, Vaughan T, Wu C-H, Xie D, et al.. BEAST 2: A Software Platform for Bayesian Evolutionary Analysis. *PLOS Computational Biology*. 2014; doi: 10.1371/journal.pcbi.1003537.

70. Rambaut A, Drummond AJ, Xie D, Baele G, Suchard MA. Posterior Summarization in Bayesian Phylogenetics Using Tracer 1.7. *Systematic Biology*. 2018; doi: 10.1093/sysbio/syy032.

71. Patterson NJ, Moorjani P, Luo Y, Mallick S, Rohland N, Zhan Y, et al.. Ancient Admixture in Human History. *Genetics*. 2012; doi: 10.1534/genetics.112.145037.

72. Maier R, Flegontov P, Flegontova O, Changmai P, Reich D. On the limits of fitting complex models of population history to genetic data. *bioRxiv*. 2022; doi: 10.1101/2022.05.08.491072.

73. Yu Y, Guan J, Xu Y, Ren F, Zhang Z, Yan J, et al.. Population-scale peach genome analyses unravel selection patterns and biochemical basis underlying fruit flavor. *Nat Commun*. Nature Publishing Group; 2021; doi: 10.1038/s41467-021-23879-2.

74. Browning BL, Browning SR. Genotype Imputation with Millions of Reference Samples. *Am J Hum Genet*. 2016; doi: 10.1016/j.ajhg.2015.11.020.

75. Browning BL, Browning SR. Improving the accuracy and efficiency of identity-by-descent detection in population data. *Genetics*. 2013; doi: 10.1534/genetics.113.150029.

76. Wu T, Hu E, Xu S, Chen M, Guo P, Dai Z, et al.. clusterProfiler 4.0: A universal enrichment tool for interpreting omics data. *The Innovation*. 2021; doi: 10.1016/j.xinn.2021.100141.

77. Chen H, Patterson N, Reich D. Population differentiation as a test for selective sweeps. *Genome research*. 2010; doi: 10.1101/gr.100545.109.

78. Zhou X, Stephens M. Efficient multivariate linear mixed model algorithms for genome-wide association studies. *Nature methods*. 2014; doi: 10.1038/nmeth.2848.

Figure legends:

Figure 1 Differences between hulled and naked oat and the selection of accessions in this study. a, Spikelet and grain of typical hulled oat (left) and naked oat (right). b, Principal component analysis (PCA) for the 189 low-depth sequencing accessions. c, Phylogenetic tree analysis for the 189 low-depth sequencing accessions. Blue indicates selected high-depth sequencing accessions. d, Geographic origin of 115 selected high-depth sequencing accessions.

Figure 2 Genetic diversity of common oat. a, The distribution of SNP density, genetic diversity and distance across chromosomes. The outer gray tracks represent the chromosomes of the OT3098 reference genome. b, The genetic diversity (π) and distance (Fst) of OH, ONL and ONC. c, The runs of homozygosity (ROH) of OH, ONL, ONC and OG. d, The linkage disequilibrium (LD) decay analysis for OH, ONL and ONC.

Figure 3 Population structure and differentiation of hulled oat and naked oat. a, Neighbor-joining (NJ) tree based on identity by stat (IBS) distance. b, A model-based ancestry estimating conducted using ADMIXTURE. c, Cross-validation error when k=2 to 8. d, Reads mapping rates of OH and ONL. e, Principal component analysis (PCA) plot of the first two principal components for all accessions. Closely related hexaploid species were excluded in this analysis. f, Divergence time of hulled oat and naked oat estimated using BEAST. The divergence time (~0.78 MYA) of A. fatua L. and cultivated oat was used as calibration.

Figure 4 Introgression analyses between OH and ONC. a, The Patterson's D statistic for testing introgression between ONC and OHc. Brown lines represent shared derived alleles, while blue

lines represent shared more derived alleles than expected due to introgression. b, The Patterson's D statistic for testing introgression between ONC and ONLc. c, The common introgressed segments (blue) across chomosomes revealed by rIBD analysis. d, Barplot of the total length of introgressed segments from different subgenomes. e and f, The GO and KEGG enrichment of the 444 introgressed genes. The plots shows the top 10 most significantly enriched terms or pathways.

Figure 5 The selective sweep analyses for local environment adaptation of ONL and improvement of ONC. a-d, The annual precipitation, annual average temperature, frost-free period, and accumulated temperature of YG and GNQ. YG represented accessions from provinces with relatively higher rainfall and temperature, and GNQ represented accessions from arid and low temperature regions of China. e, Selection signatures in GNQ genomes while using YG as reference. f-h, The spikelet number, grain number per spike, and grain weight per spike of OH, ONC, and ONL. Lowercase letters on the top of the plots indicate significant differences among groups ($P<0.01$, Student's t-test). g, Selection signatures in ONC genomes while using ONLc as reference.

Figure 6 Genetic loci associated with the smut resistance. a, Manhattan plot for the GWAS result. The horizontal dotted line depict the Bonferroni-adjusted significance threshold ($\alpha=0.05$). b, The local association signature and LD block heatmap. The red dots represent significantly associated SNPs. c, The haplotypes constructed using SNPs in the candidate gene or in 2 kb upstream of the candidate gene. d, The phenotypes of different haplotypes. Lowercase letters on the top of the plot indicate significant differences between Hap.1 and Hap. 2 ($P<0.01$, Student's t-test).

Figure 7 Genetic loci associated with the DTM. a, Manhattan plot for the GWAS result. The horizontal dotted line depict the Bonferroni-adjusted significance threshold ($\alpha=0.05$). b, The local association signature and LD block heatmap. The red dots represent significantly

associated SNPs. c, The haplotypes constructed using SNPs in the candidate gene or in 2 kb upstream of the candidate gene. d, The phenotypes of different haplotypes. Lowercase letters on the top of the plot indicate significant differences among haplotypes (P<0.01, Student's t-test).

Figure

Click here to access/download;Figure;Figures.pptx

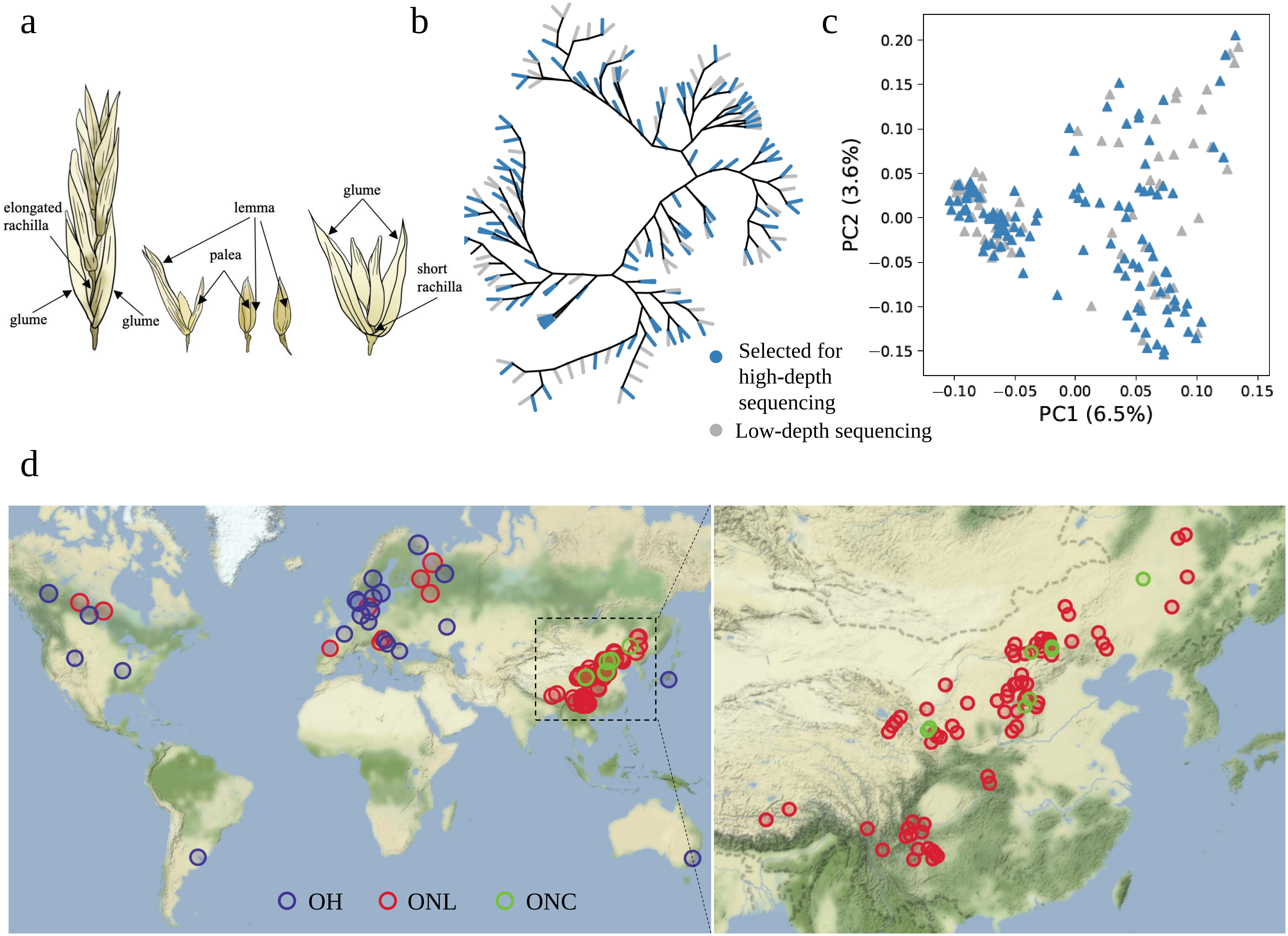

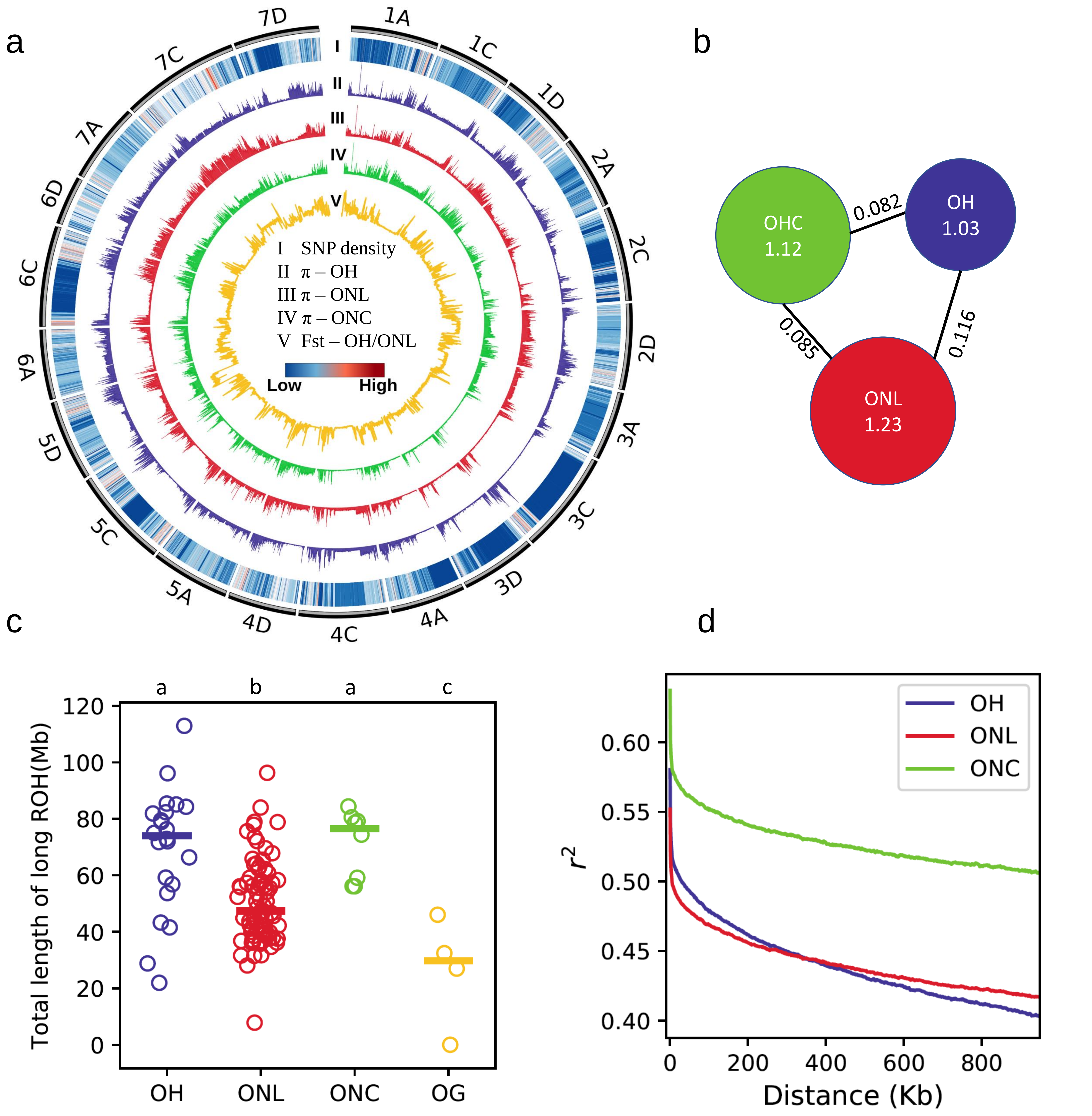

a
I SNP density
II π – OH
III π – ONL
IV π – ONC
V Fst – OH/ONL
Low
High
1A 1C 1D 2A 2C 2D 3A 3C 3D 4A 4C 4D 5A 5C 5D 6A 6C 6D 7A 7C 7D
b
OHC 1.12
OH 1.03
ONL 1.23
0.082
0.085
0.116
c
Total length of long ROH(Mb)
a b a c
OH ONL ONC OG
d
OH
ONL
ONC
r²
Distance (Kb)

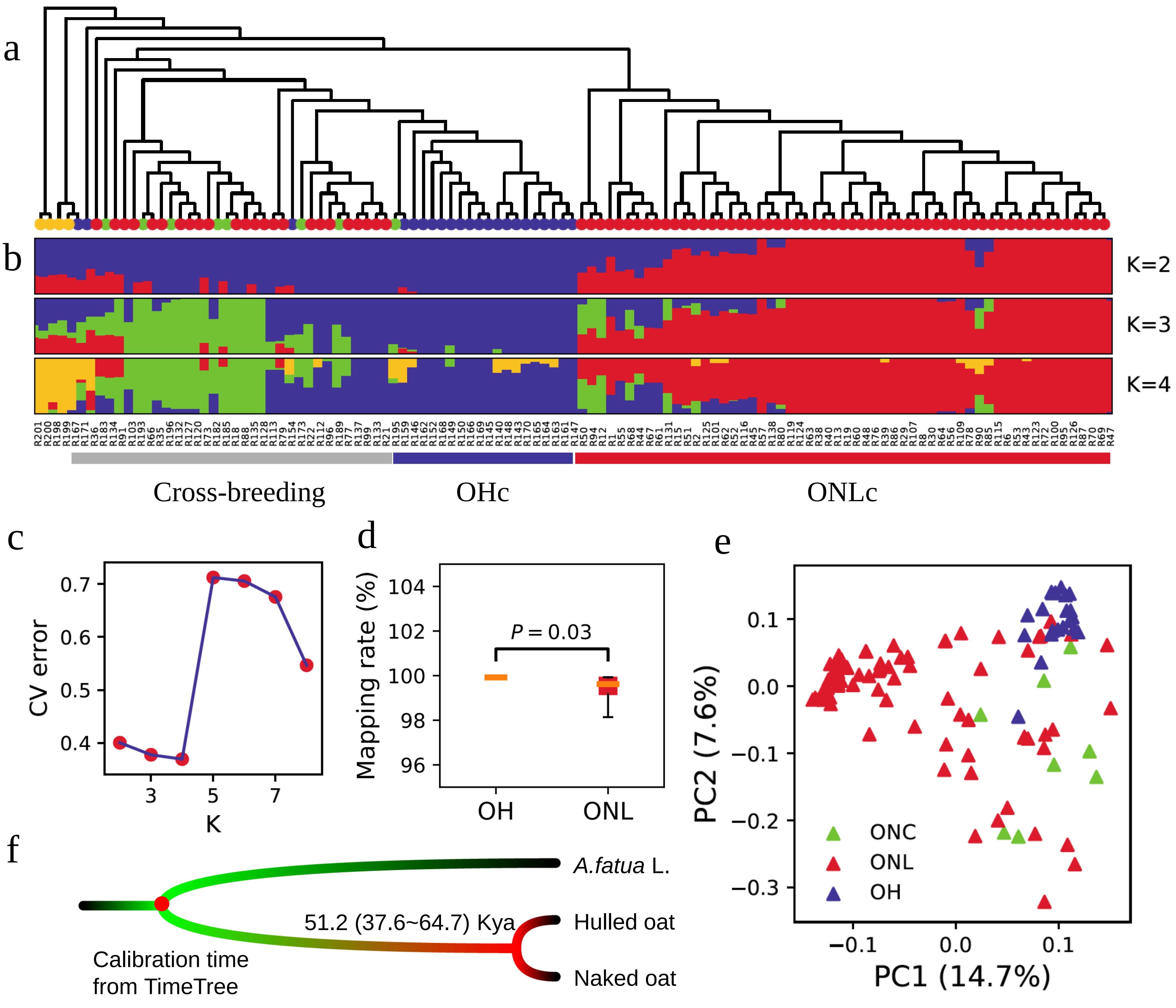
a
b
K=2
K=3
K=4
Cross-breeding
OHc
ONLc
c
CV error
0.7
0.6
0.5
0.4
3
5
7
K
d
Mapping rate (%)
104
102
100
98
96
P = 0.03
OH
ONL
e
PC2 (7.6%)
0.1
0.0
−0.1
−0.2
−0.3
ONC
ONL
OH
−0.1
0.0
0.1
PC1 (14.7%)
f
A.fatua L.
51.2 (37.6~64.7) Kya
Hulled oat
Naked oat
Calibration time
from TimeTree

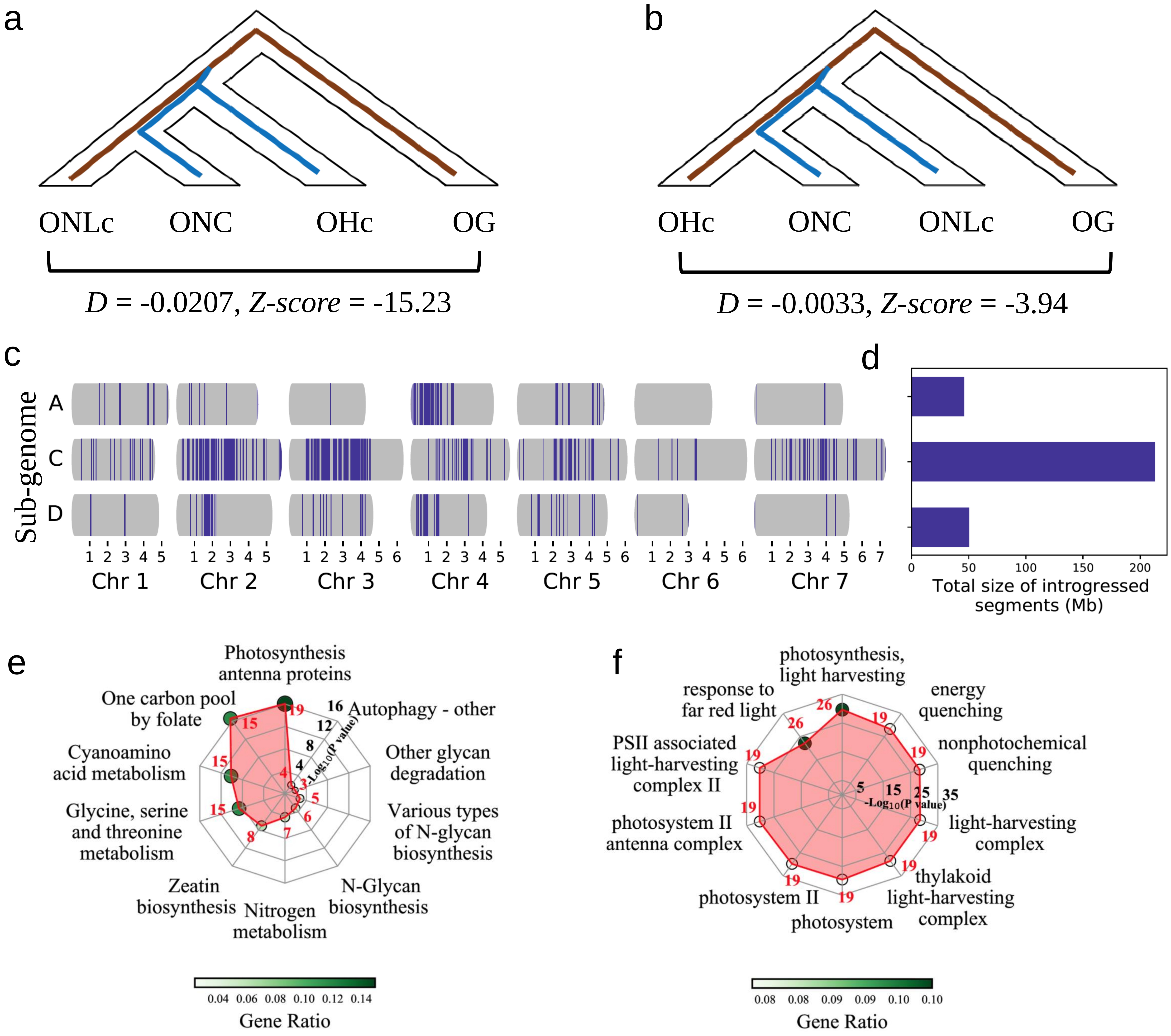

a
ONLc
ONC
OHc
OG
D = -0.0207, Z-score = -15.23
b
OHc
ONC
ONLc
OG
D = -0.0033, Z-score = -3.94
c
Sub-genome
A
C
D
Chr 1
Chr 2
Chr 3
Chr 4
Chr 5
Chr 6
Chr 7
d
0
50
100
150
200
Total size of introgressed segments (Mb)
e
Photosynthesis antenna proteins
Autophagy - other
Other glycan degradation
Various types of N-glycan biosynthesis
N-Glycan biosynthesis
Nitrogen metabolism
Zeatin biosynthesis
Glycine, serine and threonine metabolism
Cyanoamino acid metabolism
One carbon pool by folate
-Log10(P value)
Gene Ratio
0.04
0.06
0.08
0.10
0.12
0.14
f
photosynthesis, light harvesting
energy quenching
nonphotochemical quenching
light-harvesting complex
thylakoid light-harvesting complex
photosystem
photosystem II
photosystem II antenna complex
PSII associated light-harvesting complex II
response to far red light
-Log10(P value)
Gene Ratio
0.08
0.08
0.09
0.09
0.10
0.10

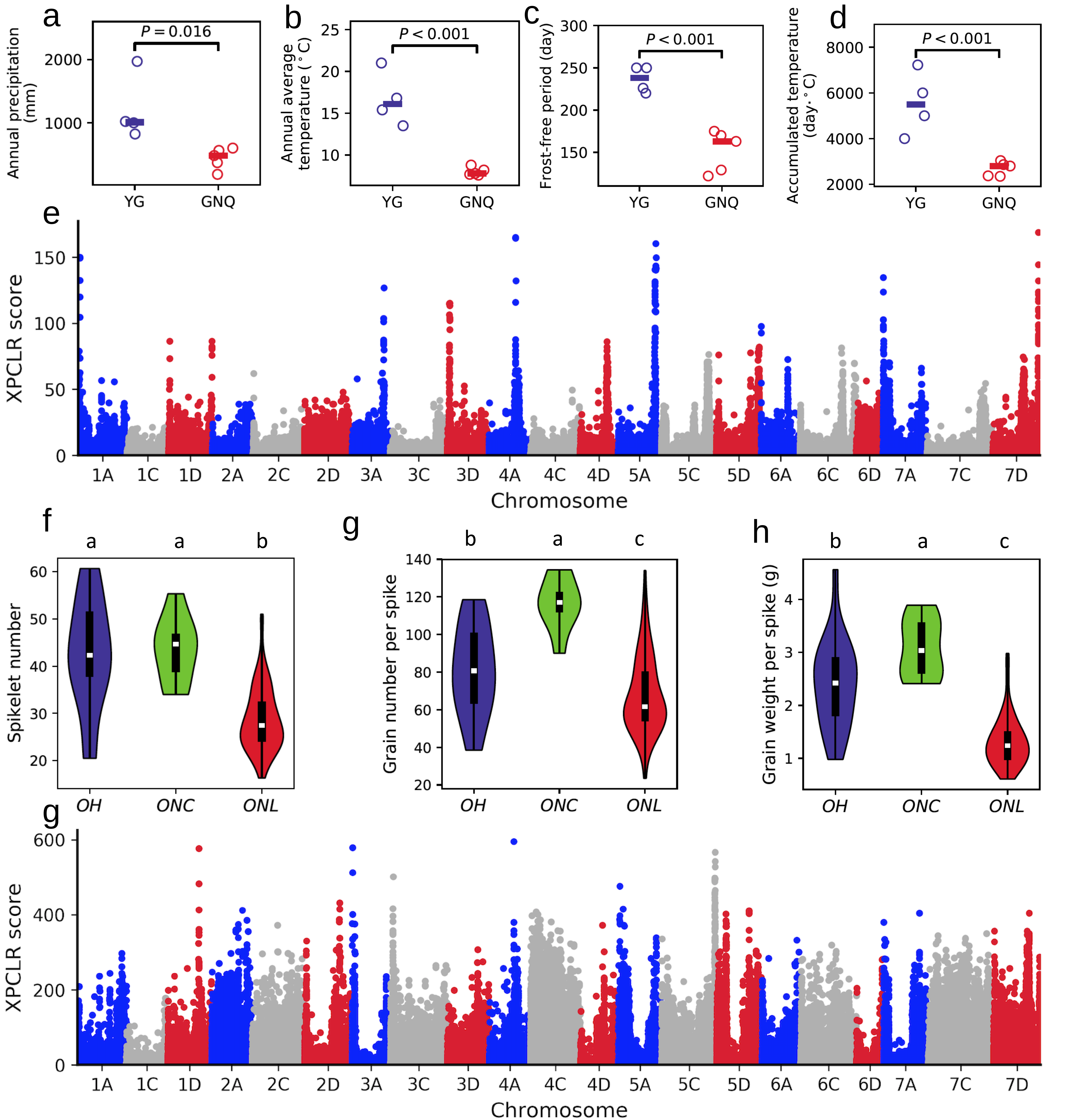
a
b
c
d
Annual precipitation (mm)
Annual average temperature (°C)
Frost-free period (day)
Accumulated temperature (day·°C)
P = 0.016
P < 0.001
P < 0.001
P < 0.001
YG
GNQ
e
XPCLR score
1A 1C 1D 2A 2C 2D 3A 3C 3D 4A 4C 4D 5A 5C 5D 6A 6C 6D 7A 7C 7D
Chromosome
f
g
h
a a b
b a c
b a c
Spikelet number
Grain number per spike
Grain weight per spike (g)
OH
ONC
ONL
g
XPCLR score
1A 1C 1D 2A 2C 2D 3A 3C 3D 4A 4C 4D 5A 5C 5D 6A 6C 6D 7A 7C 7D
Chromosome

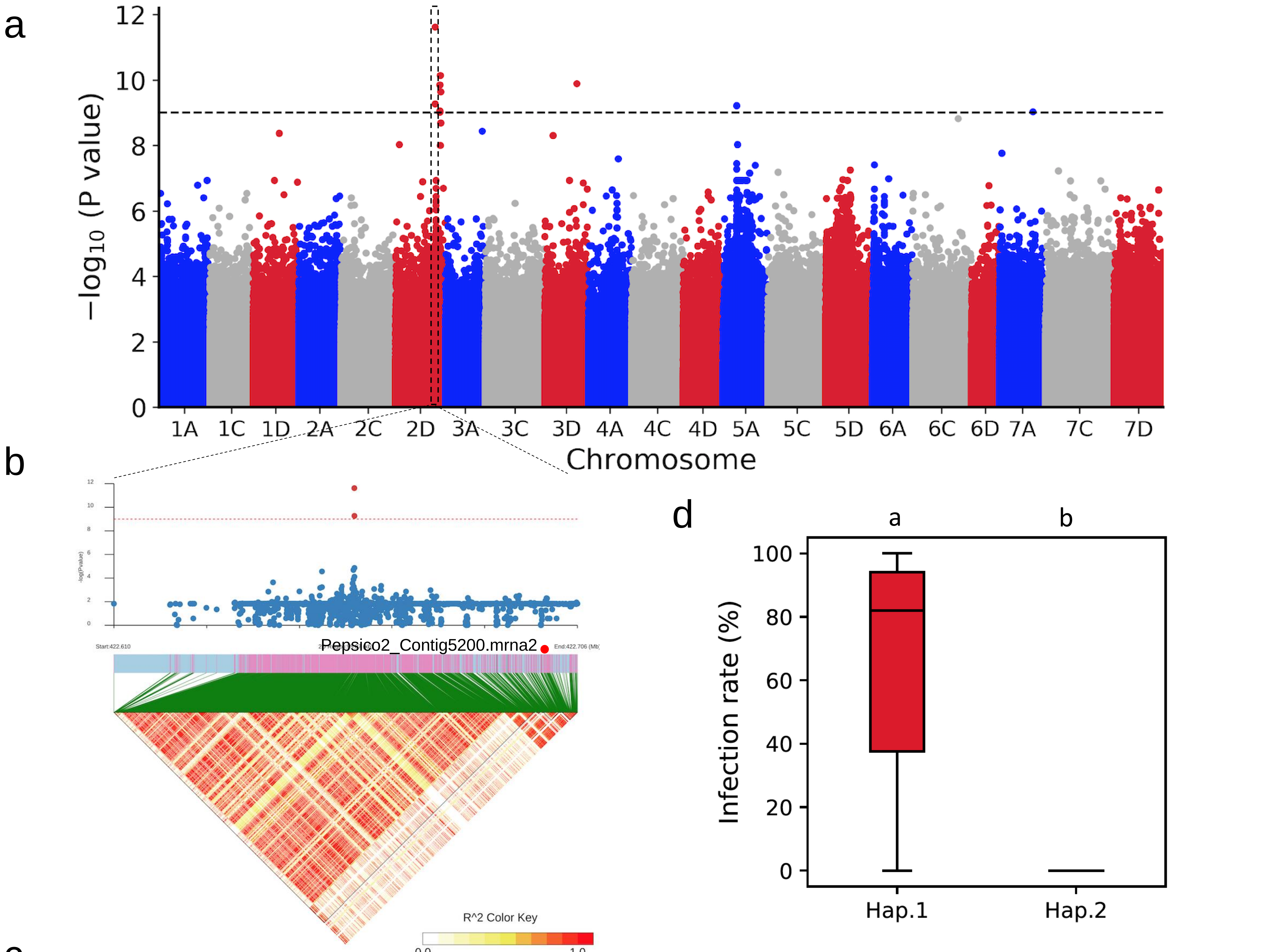

c

Effect	Upstream																										Synonymous			Intron	3' UTR	No. of accessions
Position	-1821	-1792	-1630	-1551	-1454	-1373	-1160	-1131	-958	-902	-895	-722	-714	-703	-673	-611	-564	-526	-498	-466	-453	-365	-333	-256	-217	-207	427	586	1212	975	1835	
Hap.1	G	C	T	A	G	C	A	T	C	C	T	G	G	G	A	C	G	C	T	C	A	C	G	T	A	C	G	T	C	A	C	91
Hap.2	A	T	C	G	A	T	T	C	T	T	C	A	A	A	G	G	C	T	C	T	G	T	T	C	G	G	A	C	T	T	T	12

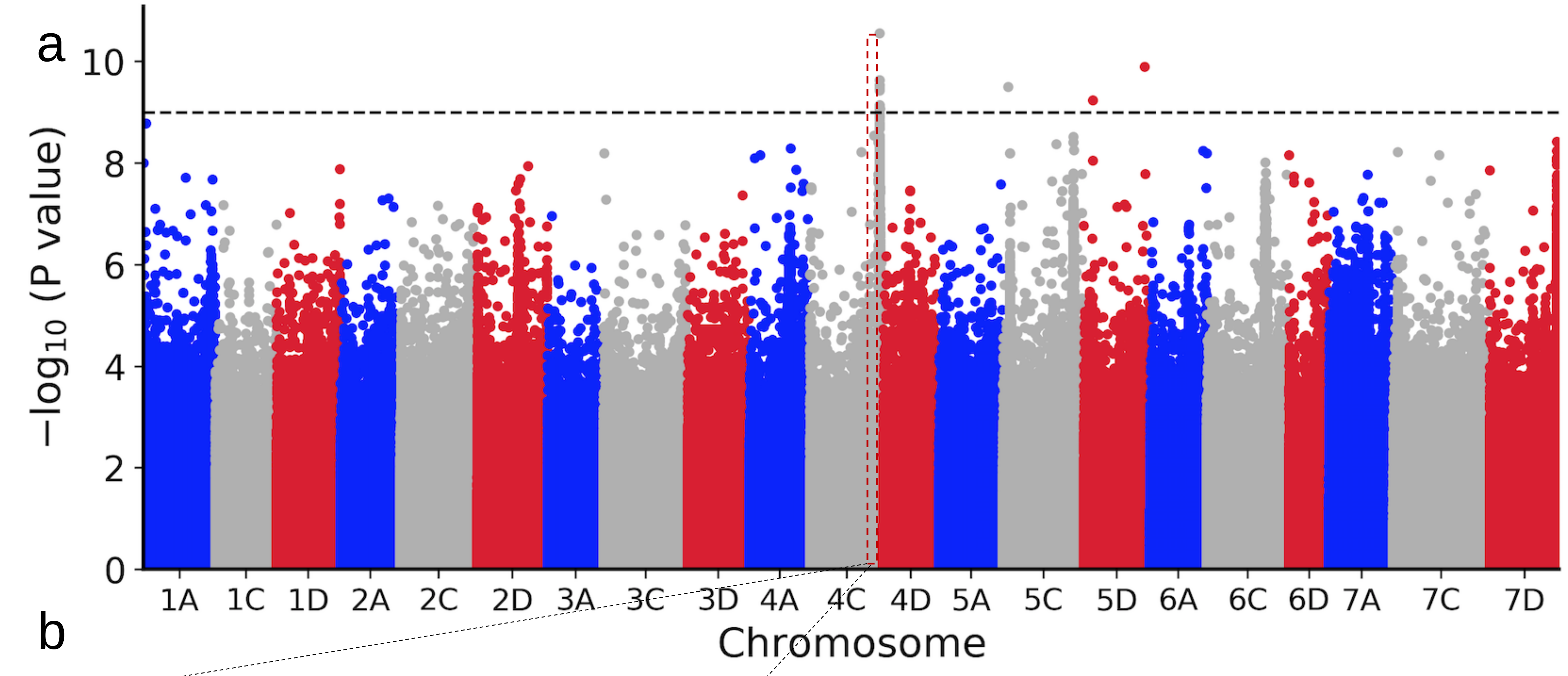

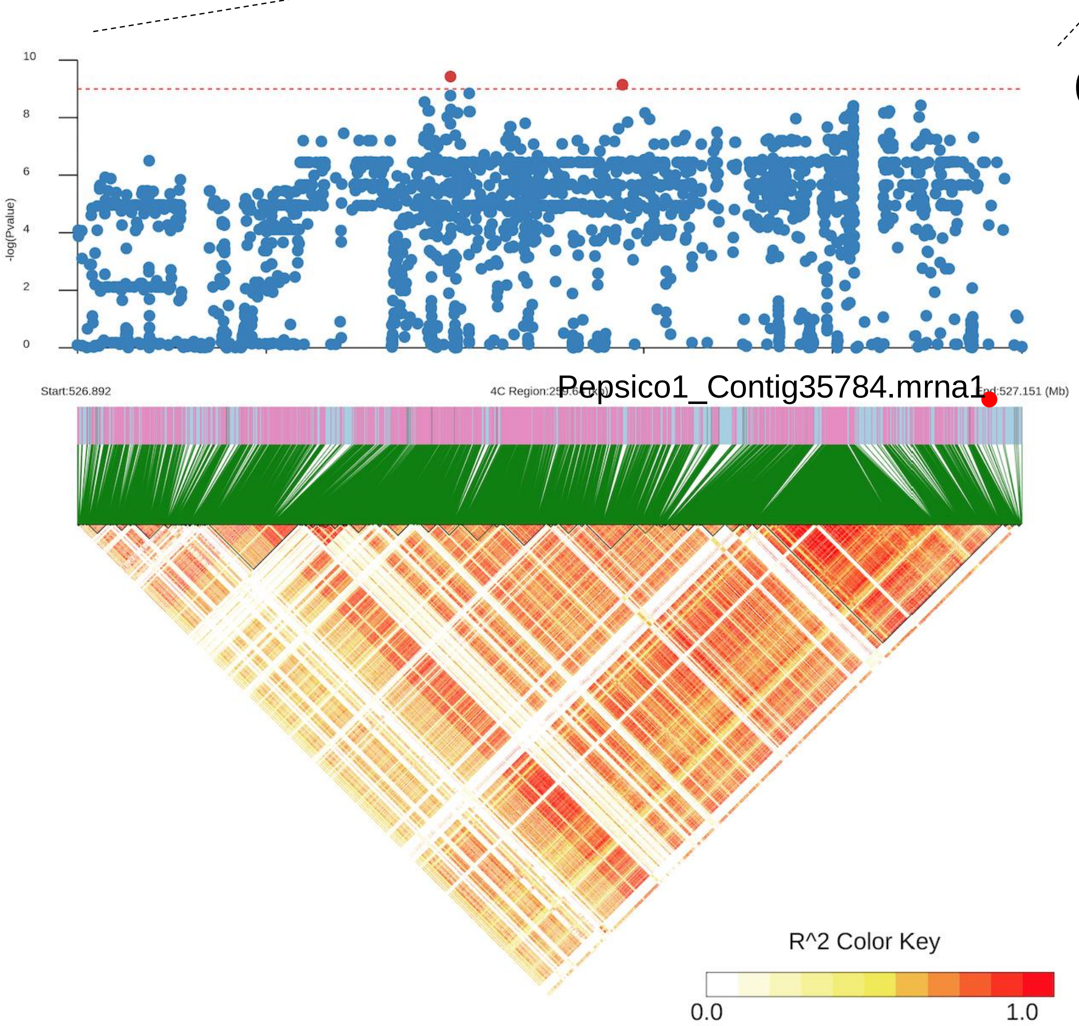

c

Effect	Upstream	5' UTR	Missense	No. of accessions
Position	-565	80	1109	
Hap.1	A	T	G	96
Hap.2	C	C	A	9
Hap.3	A	C	G	5

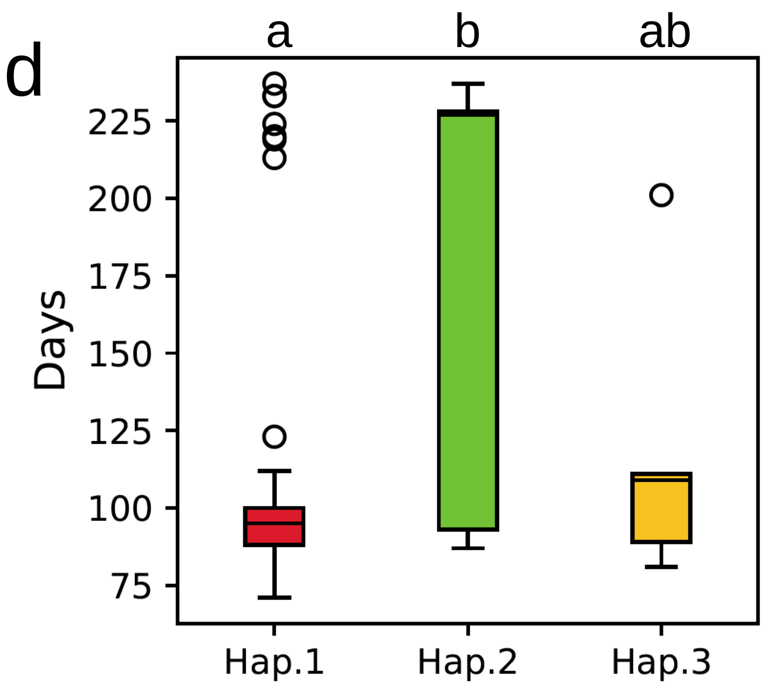

Supplement: giad061_GIGA-D-22-00306_Revision_1 [file giad061_giga-d-22-00306_revision_1.pdf]
