# Supplementary material for: Genome resequencing reveals independent domestication and breeding improvement of naked oat
Source: Gigascience. 2023 Aug 1;12:giad061. doi: 10.1093/gigascience/giad061 (PMC10390318; doi:10.1093/gigascience/giad061)
Supplement: giad061_Response_to_Reviewer_Comments_Original_Submission [file giad061_response_to_reviewer_comments_original_submission.pdf]

Author's Response To Reviewer Comments

Close

Dear Reviewers and Editors,
We appreciate your help in reviewing our work very much. All of the valuable comments and suggestions have helped improve the quality of our manuscript. Below, we have responded to the reviewers' comments point-by-point, and all corresponding improvements have been tracked and highlighted within the manuscript. Considering each author's contributions after the revised version, we have modified the order of the authors with the agreement of all authors involved. Additionally, we have adjusted the funding information supporting this work following the requirements of the funding organization.
If you have more questions, please contact us at hb_nmg@163.com
Best regards
Han Bing

Reviewer reports:

Reviewer #1: Dear Authors,

It was very fascinating to read your new genomic insights into the domestication histories of naked and hulled oats. The discovery of such a long divergence period between naked and hulled oats as well as the suggested genomic differences would add a whole new flavor to the domestication history of oats.

Looking into the data makes it clearly an interesting resource that will provide a new foundation for further analyses.
Unfortunately, you haven't ruled out any demographic effects, which lets me question your results and thus its interpretation.

Regarding the very distinct origin of your samples and the heterogenous sample sizes, demographic analysis would add robustness to your analyses, especially to get a clearer insight into the presumably secondary domestication history. We assume that the domestication history of oat is a complex one with a secondary origin from a weed. So imagine, that the weedy oat (which was already adapted to some part to the human made agricultural setting in its area of origin - SW Asia? Mediterranean?), was transported to different regions (as e.g. Eastern Asia and Central Europe) and only then followed by the final steps towards domestication? Hexaploid oats are to my knowledge fully inter-fertile and therefore cross-pollination can easily increase heterogeneity and within group diversity.

As we do assume that oat experienced a more complex domestication history I am very much looking forward to see data resources, such as what you provide here, will help to shed light into such complex histories. Nevertheless, I also think that we need to be very careful when analysing genomic data to illuminate our understanding of cultivation histories that are known to be not as straight-forward in its explanation as others (such as wheat and barley).

So regarding the origin of your samples: Since when are the different accessions maintained in the collection? Do you have insights into the collection history? How is potential cross-pollination handled?

All the best and I am very much looking forward to dive deeper into the history of oat cultivation

Response: Thank you very much for your comments.

The collection and organization of oat germplasm resources in China began in the 1950s, led by the national agricultural ministry and the Crop Science Institute of the Chinese Academy of Agricultural

Sciences. Several major collection and solicitation efforts were carried out [1].

Firstly, starting from the 1950s, relevant departments were organized by the national agricultural ministry to carry out collection and recording work nationwide. The Crop Science Institute of the Chinese Academy of Agricultural Sciences also employed dedicated personnel to collect, organize, and preserve oat germplasm resources domestically and internationally, while agricultural research units in North China successively carried out the collection and organization of local farmer varieties. By the late 1950s, the Crop Science Institute of the Chinese Academy of Agricultural Sciences imported 489 oat germplasm resources from 21 countries including the Soviet Union, Canada, Sweden, France, Denmark, Mongolia, Hungary, and Japan. By 1966, a total of 1,497 domestic and foreign oat germplasm resources were collected, organized, and registered [2].

Secondly, with the resumption of foreign introduction work in 1973, the Crop Science Institute of the Chinese Academy of Agricultural Sciences carried out the second oat germplasm resource introduction and collection work from the 1970s to the 1990s. According to the "Catalogue of Chinese Oat Germplasm Resources I" [3] compiled in the 1980s and the "Catalogue of Chinese Oat Germplasm Resources II" [4] compiled in the 1990s, a total of 2,978 oat resources were included, covering nine species of oat. At the beginning of the 1980s, three levels of seed banks were established at the national, regional, and major producing province (region) levels in China. At the same time, wild oat resources in North China, Northwest China, Southwest China, and Tibet were investigated and identified.

Thirdly, since the 1990s, with the increasing attention to oat research and strengthened national cooperation, introduction and collection work has been carried out gradually. Through cooperation with the Plant Gene Resources of Canada (PGRC), wild oat and local variety resources have been collected from around the world. Currently, China has collected and preserved over 6,200 oat germplasm resources, including 29 species [5].

Oats are a typical self-pollinating plant that generally undergoes self-fertilization in the absence of human intervention, but natural hybridization exists between different materials [6-8]. The structure of oat flowers determines its low natural outcrossing rate. We applied for the most primitive preserved seeds from the germplasm bank, which were very limited in quantity and needed to be multiplied. To prevent contamination and natural hybridization during propagation, the single seed descents (SSD) method was used, where one seed is grown, protected from out-crossing, and then one seed from the progeny is regrown, and this process is repeated for several generations until enough seeds are obtained for molecular experiments and phenotypic studies.

We completely agree with your viewpoint that demographic history influences the inference of genetic diversity, divergence time, and other conclusions. However, due to the limited data currently available, we are unable to draw more in-depth conclusions about the domestication history of oats. For example, to study gene flow, we need more outgroup genomic information; To infer population demographic history, we require larger-scale population resequencing data for accurate SNP phasing (We have performed MSMC analysis on several hulled and naked oat genomes with high sequencing depth, but the results, whether for all chromosomes or for each of the three subgenomes separately, did not yield any meaningful information.). We have already begun to de novo assemble genomes of several representative hexaploid related species of oats and representative hulled and naked oats, as well as conducting larger-scale whole-genome resequencing. We hope that with these data, we can gain a more thorough understanding of the domestication history of oats. Accordingly, we have made relevant explanations in the Discussion section (Line 341-352). Thank you for your valuable suggestions.

[1] Zheng, D., and Zhang, Z. (2017). Introduction and Utilization of Foreign Oat Germplasm Resources in China. Journal of Plant Genetic Resources 18, 1001–1005. doi: 10.13430/j.cnki.jpgr.2017.06.001.
[2] Zhao, X., Rong, Y., and Zhao, L. (2007). Collection and Evaluation of Oat Germplasm Resources in China. Pratacultural Science 24, 36–40.
[3] Crop Germplasm Resources Institute, Chinese Academy of Agricultural Sciences (1984). Catalogue of Chinese Oat Germplasm Resources I. China Agriculture Press, 1-97.
[4] Crop Germplasm Resources Institute, Chinese Academy of Agricultural Sciences (1996). Catalogue of Chinese Oat Germplasm Resources I. China Agriculture Press, 1-79.

[5] Peng, Y. (2019). Oat Germplasm Resources and Breeding and Cultivation Management in Southwest China. Chinese Agriculture Press, 17-18.
[6] Shorter, R., Gibson, P., and Frey, K. J. (1978). Outscoring Rates in Oat Species Crosses (Avena sativa L. × A. sterilis L.)1. Crop Science 18:877-878, cropsci1978.0011183X001800050051x. doi: 10.2135/cropsci1978.0011183X001800050051x.
[7] Grindeland, R. L., and Frohberg, R. C. (1966). Outcrossing of Oat Plants (Avena sativa L.) Grown from Mutagen-Treated Seeds1. Crop Science 6, cropsci1966.0011183X000600040031x. doi: 10.2135/cropsci1966.0011183X000600040031x.
[8] Yang, F., and Wang, Z. (2021). Genetic Diversity Analysis and Outcrossing Rate Estimation of Oat Based on SSR Molecular Markers. Molecular Plant Breeding.

Reviewer #2: 1. There are more We word or We also words in the manuscript. As the typical original research article, passive voice is the main expression model, it will be more accurate and smoother after revised the We word in the whole manuscript, especially in the Abstract.

Response: Thank you very much for your kind suggestion. We have modified most of the active voice into passive voice.

2. Line84-85 and line 101-102, 325 is repetition.

Response: The repetitive sentences have been modified.

3. The Latin names should be added in the bracket after hulled oat and naked oat while they present firstly.

Response: Thank you very much for your professional advice. We have made the modifications as requested.

4. The Avena nuda is the homotypic synonym of A.sativa subsp.nuda or A.sativa var.nuda, and the reference genome OT3098 is also from a species of A.sativa, the Latin name should be present in the manuscript.

Response: We have made the modifications as requested.

5. As the allohexaploid oat, its domestication is complicated, especially for that the evolution and differentiation of subgenomes might inconsistent. So the divergence times in Fig3f should be re-estimated based on Sub-genome A, Sub-genome C, Sub-genome D, respectively. The Kya in Fig3f could be replaced by Mya.
Response: Thank you for your suggestion. We have separately calculated the divergence time of the three subgenomes. The divergence time of subgenome A is 47.3 (34.6~59.8) Kya, subgenome C is 47.0 (32.9~62.1) Kya, and subgenome D is 53.3 (38.8~67.5) Kya. We also included this result in our manuscript. We confirmed again that the time unit is indeed Kya.

6. The sequence depth of three germplasms (R21, R22, R86) selected were above 20X, estimation of the effective population size (Ne) history by using PSMC or MSMC2 as supplementary figure might provide another evidence of naked oat domestication independently.

Response: Thank you for your suggestion. We performed MSMC analysis on several hulled and naked oat genomes with high sequencing depth, but the results, whether for all chromosomes or for each of the three subgenomes separately, did not yield any meaningful information. We are unsure if this is due to MSMC not being effective in polyploid species such as oat or for other reasons, and have therefore decided not to include these results in the manuscript.

7. The means of brown and blue lines in Fig4a and Fig4b should be list in the legend.

Response: Thanks for your comment. We have added the statement "Brown lines represent shared derived alleles, while blue lines represent shared more derived alleles than expected due to introgression." to the figure legend.

8. The text and figures in results of GWAS was inconsistent.

Response: Thank you for reminding us. We are ashamed of our carelessness. The necessary corrections have been made.

9. The results of significance analysis should be list as label, especially for the histogram in Fig2c, Fig3d, Fig5f-5h, Fig6d, Fig7d.

Response: Thank you for your comment. Significant labels have now been added to Fig2c and Fig3d. Fig5f-5h, Fig6d, and Fig7d already had significant labels as they involve comparing more than three groups, so they are marked with letter notation at the top of the figures.

10. The source of seeds should be definite in the plant materials of Materials and Methods. The 115 accessions in the manuscript were suggested to replace as 115 germplasms after the seed was provided by NGBC or theses germplasms had accessed as in NGBC, it might be misunderstood as these data were download from public database by the accessions were overmentioned.

Response: Thank you for your comment. We have added an explanation about the source of our seeds. However, we still use "accession" to represent different oat varieties because it not only denotes the registration number of the sequencing data but also identifies the germplasm resources in the germplasm bank. We believe that this will not cause any confusion, as in many other publications (e.g., [1-3]), accession is also used to represent different varieties, even for data generated by the studies themselves.

1. Cai, X., Sun, X., Xu, C., Sun, H., Wang, X., Ge, C., Zhang, Z., Wang, Q., Fei, Z., Jiao, C., et al. (2021). Genomic analyses provide insights into spinach domestication and the genetic basis of agronomic traits. Nat Commun 12, 7246. 10.1038/s41467-021-27432-z.
2. Yu, Y., Guan, J., Xu, Y., Ren, F., Zhang, Z., Yan, J., Fu, J., Guo, J., Shen, Z., Zhao, J., et al. (2021). Population-scale peach genome analyses unravel selection patterns and biochemical basis underlying fruit flavor. Nat Commun 12, 3604. 10.1038/s41467-021-23879-2.
3. Liu, Y., Du, H., Li, P., Shen, Y., Peng, H., Liu, S., Zhou, G.-A., Zhang, H., Liu, Z., Shi, M., et al. (2020). Pan-Genome of Wild and Cultivated Soybeans. Cell. 10.1016/j.cell.2020.05.023.

11. The heatmap of SNP density in Fig2A was suggested to present as single color with 3 gradients, such as blues-3-seq or greens-3-seq. The π= or Fst= was suggest to add before the numbers.

Response: Thank you for your comment. We have followed your suggestion and added "π=" and "Fst=" before the numbers. However, we insist on using the original colors in the heatmap because the color scheme from cool (blue) to warm (red) is the most commonly used color scheme for heatmaps.

Close
