# Supplementary material for: Genome resequencing reveals independent domestication and breeding improvement of naked oat
Source: Gigascience. 2023 Aug 1;12:giad061. doi: 10.1093/gigascience/giad061 (PMC10390318; doi:10.1093/gigascience/giad061)
Supplement: giad061_Response_to_Reviewer_Comments_Revision_1 [file giad061_response_to_reviewer_comments_revision_1.pdf]

Author's Response To Reviewer Comments

Close

Dear Reviewers and Editors,
Thank you again for your valuable revision suggestions. Below, we have responded to the reviewers' comments point-by-point, and all corresponding improvements have been tracked and highlighted within the manuscript.
If you have more questions, please contact us at hb_nmg@163.com
Best regards,
Han Bing

Reviewer reports:

Reviewer #2: 1. The response of comment 6 is not unambiguous. What means is about not yield any meaningfull information? Could not get the final Figure or the final figure contained the result not suggesting the conclusion? The software of PSMC is recommended priorly if final figure could not be obtained and the Beta-PSMC and MSMC2 could be also tried if the PSMC were no available.
Estimation of effective population size (Ne) history is still recommended. Only three samples sequencing depth above 20 X is available for this analysis and R21，R22 and R86 could be analyzed respectively with PSMC. The final result or procedure files should be presented as the supplementary or attachment in the next revision whether it is meaningful or not.
2. The conclusion in the abstract of the previous speculation that naked oat was a variant of hulled oat was rejected is dubious or confused. This verdict is also a suggestion even the effective population size (Ne) history above were added in the next revision according to that the sample of sequence depth above 20 X is few.

Response: Thank you again for your rigorous and responsible review. Your comments have greatly improved the quality of our paper. We apologize for not being able to upload the MSMC resulting images in our last response to your comments. This time we have included the MSMC results in the supplementary material and added relevant content in the Discussion and Method sections. The added content was also attached below:

L288-292:
Furthermore, PSMC' (a special case of MSMC for two haplotypes) was used to calculate the demographic history. We found that regardless of whether all chromosomes or three subsets of subgenomes were calculated separately, the effective population size changes of hulled and naked oat were very similar: they have been decreasing since one million years ago until now. Compared with hulled oat, naked oat did not experience a more severe bottleneck (Figure S1).

L396-410:
Demographic history
To infer demographic history, MSMC2 [71] was used. To ensure reliability, nine accessions (R57, R60, R67, R70, R86 for naked oat and R148, R164, R169 and R170 for hulled oat) without admixture between naked oat and hulled oat (indicated by ADMIXTURE and TREE) were selected for analysis with a sequencing depth of more than 15X. Genome regions were masked when the coverage depth was below 15× after removing reads with mapping quality <20, and were also masked using Heng Li's SNPable tool (http://lh3lh3.users.sourceforge.net/snpable.shtml). In brief, the reference genome was split into overlapping 35-mers and then mapped back to the reference genome using BWA (bwa aln -R 1000000 -O 3 -E 3). Only regions of the majority of overlapping 35-mers were mapped back uniquely and without 1-mismatch were kept. Scaled times were converted to years assuming a generation time of 1 year and a mutation rate of 6.5 × 10−9 per site per generation. The atmospheric surface air temperature relative to the present (°C) and the ice volume contribution to the marine isotope signal (relative to the present) were

downloaded from the NCDC database (https://www.ncdc.noaa.gov).

L735-739:
Figure S1 Demographic history inferred using MSMC2. Nine accessions (R57, R60, R67, R70, R86 for naked oat and R148, R164, R169 and R170 for hulled oat) without admixture between naked oat and hulled oat (indicated by ADMIXTURE and TREE) were selected for analysis. The ice volume and global surface air temperature, as reconstructed using benthic oxygen isotope stack, were shown as gray solid and dotted lines respectively.

Close
