# Supplementary material for: Genome resequencing reveals independent domestication and breeding improvement of naked oat
Source: Gigascience. 2023 Aug 1;12:giad061. doi: 10.1093/gigascience/giad061 (PMC10390318; doi:10.1093/gigascience/giad061)
Supplement: giad061_Reviewer_2_Report_Original_Submission — Xupo Ding -- 2/6/2023 Reviewed [file giad061_reviewer_2_report_original_submission.pdf]

Reviewer Report

Title: Genome resequencing reveals independent domestication and breeding improvement of naked oat

Version: Original Submission **Date:** 2/6/2023

Reviewer name: Xupo Ding

Reviewer Comments to Author:

1. There are more We word or We also words in the manuscript. As the typical original research article, passive voice is the main expression model, it will be more accurate and smoother after revised the We word in the whole manuscript, especially in the Abstract.
2. Line84-85 and line 101-102, 325 is repetition.
3. The Latin names should be added in the bracket after hulled oat and naked oat while they present firstly.
4. The Avena nuda is the homotypic synonym of A.sativa subsp.nuda or A.sativa var.nuda, and the reference genome OT3098 is also from a species of A.sativa, the Latin name should be present in the manuscript.
5. As the allohexaploid oat, its domestication is complicated, especially for that the evolution and differentiation of subgenomes might inconsistent. So the divergence times in Fig3f should be re-estimated based on Sub-genome A, Sub-genome C, Sub-genome D, respectively. The Kya in Fig3f could be replaced by Mya.
6. The sequence depth of three germplasms (R21, R22, R86) selected were above 20X, estimation of the effective population size (Ne) history by using PSMC or MSMC2 as supplementary figure might provide another evidence of naked oat domestication independently.
7. The means of brown and blue lines in Fig4a and Fig4b should be list in the legend.
8. The text and figures in results of GWAS was inconsistent.
9. The results of significance analysis should be list as label, especially for the histogram in Fig2c, Fig3d, Fig5f-5h, Fig6d, Fig7d.
10. The source of seeds should be definite in the plant materials of Materials and Methods. The 115 accessions in the manuscript were suggested to replace as 115 germplasms after the seed was provided by NGBC or theses germplasms had accessed as in NGBC, it might be misunderstood as these data were download from public database by the accessions were overmentioned.
11. The heatmap of SNP density in Fig2A was suggested to present as single color with 3 gradients, such as blues-3-seq or greens-3-seq. The Ï€= or Fst= was suggest to add before the numbers.

Methods

Are the methods appropriate to the aims of the study, are they well described, and are necessary controls included? Choose an item.

Conclusions

Are the conclusions adequately supported by the data shown? Choose an item.

Reporting Standards

Does the manuscript adhere to the journal's guidelines on minimum standards of reporting? Choose an item.

Choose an item.

Statistics

Are you able to assess all statistics in the manuscript, including the appropriateness of statistical tests used? Choose an item.

Quality of Written English

Please indicate the quality of language in the manuscript: Choose an item.

Declaration of Competing Interests

Please complete a declaration of competing interests, considering the following questions:

- Have you in the past five years received reimbursements, fees, funding, or salary from an organisation that may in any way gain or lose financially from the publication of this manuscript, either now or in the future?
- Do you hold any stocks or shares in an organisation that may in any way gain or lose financially from the publication of this manuscript, either now or in the future?
- Do you hold or are you currently applying for any patents relating to the content of the manuscript?
- Have you received reimbursements, fees, funding, or salary from an organization that holds or has applied for patents relating to the content of the manuscript?
- Do you have any other financial competing interests?
- Do you have any non-financial competing interests in relation to this paper?

If you can answer no to all of the above, write 'I declare that I have no competing interests' below. If your reply is yes to any, please give details below.

'I declare that I have no competing interests.

I agree to the open peer review policy of the journal. I understand that my name will be included on my report to the authors and, if the manuscript is accepted for publication, my named report including any attachments I upload will be posted on the website along with the authors' responses. I agree for my

report to be made available under an Open Access Creative Commons CC-BY license (http://creativecommons.org/licenses/by/4.0/). I understand that any comments which I do not wish to be included in my named report can be included as confidential comments to the editors, which will not be published.

Choose an item.

To further support our reviewers, we have joined with Publons, where you can gain additional credit to further highlight your hard work (see: https://publons.com/journal/530/gigascience). On publication of this paper, your review will be automatically added to Publons, you can then choose whether or not to claim your Publons credit. I understand this statement.

Yes Choose an item.
