# Supplementary material for: Genome resequencing reveals independent domestication and breeding improvement of naked oat
Source: Gigascience. 2023 Aug 1;12:giad061. doi: 10.1093/gigascience/giad061 (PMC10390318; doi:10.1093/gigascience/giad061)
Supplement: giad061_Reviewer_2_Report_Revision_1 — Xupo Ding -- 5/28/2023 Reviewed [file giad061_reviewer_2_report_revision_1.pdf]

Reviewer Report

Title: Genome resequencing reveals independent domestication and breeding improvement of naked oat

Version: Revision 1 **Date:** 5/28/2023

Reviewer name: Xupo Ding

Reviewer Comments to Author:

1. The response of comment 6 is not unambiguous. What means is about not yield any meaningfull information? Could not get the final Figure or the final figure contained the result not suggesting the conclusion? The software of PSMC is recommended priorly if final figure could not be obtained and the Beta-PSMC and MSMC2 could be also tried if the PSMC were no available.
Estimation of effective population size (Ne) history is still recommended. Only three samples sequencing depth above 20 X is available for this analysis and R21ï¼ŒR22 and R86 could be analyzed respectively with PSMC. The final result or procedure files should be presented as the supplementary or attachment in the next revision whether it is meaningful or not.
2. The conclusion in the abstract of the previous speculation that naked oat was a variant of hulled oat was rejected is dubious or confused. This verdict is also a suggestion even the effective population size (Ne) history above were added in the next revision according to that the sample of sequence depth above 20 X is few.

Methods

Are the methods appropriate to the aims of the study, are they well described, and are necessary controls included? Choose an item.

Conclusions

Are the conclusions adequately supported by the data shown? Choose an item.

Reporting Standards

Does the manuscript adhere to the journal's guidelines on minimum standards of reporting? Choose an item.

Choose an item.

Statistics

Are you able to assess all statistics in the manuscript, including the appropriateness of statistical tests used? Choose an item.

Quality of Written English

Please indicate the quality of language in the manuscript: Choose an item.

Declaration of Competing Interests

Please complete a declaration of competing interests, considering the following questions:

- Have you in the past five years received reimbursements, fees, funding, or salary from an organisation that may in any way gain or lose financially from the publication of this manuscript, either now or in the future?
- Do you hold any stocks or shares in an organisation that may in any way gain or lose financially from the publication of this manuscript, either now or in the future?
- Do you hold or are you currently applying for any patents relating to the content of the manuscript?
- Have you received reimbursements, fees, funding, or salary from an organization that holds or has applied for patents relating to the content of the manuscript?
- Do you have any other financial competing interests?
- Do you have any non-financial competing interests in relation to this paper?

If you can answer no to all of the above, write 'I declare that I have no competing interests' below. If your reply is yes to any, please give details below.

I declare that I have no competing interests

I agree to the open peer review policy of the journal. I understand that my name will be included on my report to the authors and, if the manuscript is accepted for publication, my named report including any attachments I upload will be posted on the website along with the authors' responses. I agree for my report to be made available under an Open Access Creative Commons CC-BY license (http://creativecommons.org/licenses/by/4.0/). I understand that any comments which I do not wish to be included in my named report can be included as confidential comments to the editors, which will not be published.

Choose an item.

To further support our reviewers, we have joined with Publons, where you can gain additional credit to further highlight your hard work (see: https://publons.com/journal/530/gigascience). On publication of this paper, your review will be automatically added to Publons, you can then choose whether or not to claim your Publons credit. I understand this statement.

Yes Choose an item.
